# Gravity-induced seismicity modulation on planetary bodies and their natural satellites

**DOI:** 10.1038/s41598-024-52809-7

**Published:** 2024-01-28

**Authors:** Batakrushna Senapati, Bhaskar Kundu, Birendra Jha, Shuanggen Jin

**Affiliations:** 1 Department of Earth and Atmospheric Sciences, NIT Rourkela, Rourkela, 769008 India; 2 Department of Chemical Engineering and Materials Science, University of Southern California, Los Angeles, CA 90007-1211 USA; 3 School of Surveying and Land Information Engineering, Henan Polytechnic University, Jiaozuo, 454000 China; 4 Shanghai Astronomical Observatory, Chinese Academy of Sciences, Shanghai, 200030 China

**Keywords:** Planetary science, Geodynamics, Seismology, Tectonics

## Abstract

Ground-based monitoring of seismicity and modulation by external forces in the field of planetary seismology remains equivocal due to the lack of natural observations. Constrained by the natural observations (including Earthquakes, Moonquakes, and Marsquakes) and theoretical models, we present the variation in gravitational acceleration “g” of different solar system objects, combined with external harmonic forcings that are responsible for seismicity modulation on the planetary bodies and their natural satellites. From the global diversity in seismicity modulation, it has been observed that the plate-boundary regions on the Earth exhibit both short and long-period seismicity modulation. In contrast, the stable plate interior regions appear to be more sensitive to long-period seismicity modulation, however, lacking in short-period modulation. The deep Moonquakes are susceptible for both the lunar tidal period (13.6 days and 27 days) and long-period pole wobble modulation (206 days), whereas shallow emergent type moonquakes show a seismic periodicity at the lunation period (29.5 days). Further, the seasonal variation with an annual seismicity burst and seismic periodicity at polar wobble periods for high-frequency Marsquakes captured by InSight lander indicate a natural origin. Whereas diurnal and semi-diurnal periodicity along with Phobos’ tidal period, indicate possible artifacts due to different detection probabilities and non-seismic noise in the Martian environment. We argue that, in the context of rate-state-dependent fault friction, the gravity-induced resonance destabilization model appears to be better agreement with the contrast and relative diversity in seismicity modulation linked to the Earth, Moon, and Mars.

## Introduction

Out of the four fundamental physical forces of nature (i.e. Nuclear strong force, Electromagnetic force, Nuclear weak force, and Gravitational force), gravity is the weakest force, but probably the most intuitive and familiar force of the four. However, its existence in the universe has been realized to be one of the most challenging to explain. Sir Isaac Newton was the first to propose the universal law of gravitation in 1665^1^, which eventually set the platform for understanding planetary bodies’ motion and their natural satellites. It also relates to periodic deformation on the planetary surface and ocean world, exerted by the gravitational attraction from their satellites and the Sun during rotational cycles in their specific orbits.

The tidal modulation of seismic activity in the different planets and their satellites, especially for the Earth-Moon-Sun system, is fairly well-constrained^2–5^. The shallow and deep moonquakes have been linked with the tidal deformation of Earth^6–8^. Further, the Moon’s tides also influence the tidal modulation of earthquakes in a diverse range of tectonic settings on the Earth, including mid-ocean ridges^9,10^, volcanic systems^11^, subduction zones^4,12^ and deep-seated non-volcanic tremor zones^13–16^. Moreover, tidally-modulated Marsquakes (i.e. induced by Phobos tides) due to pore pressure buildup from the cooling of the Martian surface also has been theoretically hypothesized^17^. However, the recorded High Frequency (HF) Marsquakes observed by NASA’s InSight (Interior exploration using Seismic Investigations, Geodesy and Heat Transport) mission has suggested no such tidal periodicity related to Phobos’ orbit^18^. It has been argued that the tidal interaction between planets and their satellites that orbit them, dissipates heat energy from their interior, and a fraction of that energy can be released in the form of seismic energy^19^. This tidally-induced heat energy dissipation model has predicted that many moons in the Solar System (e.g. Io, Europa, Titan) and exoplanetary bodies (e.g. TRAPPIST 1b, 1c, 1e; Kepler 20e, 20f.; HD 219134b) exhibit more seismic activity than the Earth’s moon^19^.

There are several other forces that are also capable of modulating the seismicity on the Earth. This includes hydrological loading, surface ice/snow loading, reservoir water level fluctuations, glacial isostatic rebound, atmospheric loading, thermo-elastic loading, sediment unloading, pole tide, and pole wobble, etc.^20–27^. The seismicity associated with the New Madrid seismic zone, USA, Non-volcanic tremor in the Cascadia subduction zone and mid-crustal seismicity in the Nepal Himalayan exhibit annual seismicity modulation by the hydrological load-induced stress^28–30^. In contrast, the annual modulation of seismicity observed in the Koyana-Warna region, India, is caused by the variation of the reservoir water level^31^. Similarly, the annual modulation of seismicity was also observed in the Marsquakes due to seasonal variation with a peak rate during the Martian summer time^18^, but no such seasonal modulation of seismicity was reported in the case of Moonquakes. However, ground-based monitoring of seismicity and triggering (or modulation) capability remains poorly constrained due to the lack of natural observations.

In fact, all the above mentioned seismic activities on different planetary bodies, exoplanets, and their natural satellites have been linked either with their orbital motion, seasonal variation of climatic factors, periodic tidal deformations exerted by the gravitational attraction, or the tidally-induced heat energy dissipation model. However, there are several caveats remain regarding the effect of gravity on seismicity modulation:

(i) There is no unified model/overview provided for the impact of gravity on the seismicity modulation of the planetary objects and their satellites based on the observational, theoretical and mechanical framework in the context of rate-state-dependent friction, to explain the discrepancy between periodic seismicity modulation at different shorter-period (e.g. semi-diurnal, diurnal, fortnight and other tidal constituents) and long-period (e.g. semi-annual, annual, pole tide, pole wobble, multi-annual) time scales.
(ii) It has been observed that relatively faster-moving plate boundaries on the Earth are susceptible to both shorter-period and long-period seismicity modulation in response to external stress perturbation. In contrast, diffuse deformation regions and stable plate interiors appear to be more sensitive to long-period seismicity modulation and less sensitive to short-period modulation^32^. Do we expect identical characteristics for Marsquakes or Moonquakes, or other Planetary bodies?
(iii) The existence of solar diurnal tide, semi-diurnal tide, or tidal periodicity related to Phobos’ orbit in the HF Marsquakes remains equivocal in the context of observations and gravity-controlled mechanical framework.
(iv) The deep moonquakes (DMQ) originated at ~ 700–1200 km depth recorded by the Apollo Passive Seismic Experiments, and their relationship with the periodic tidal forcing has been a well-established fact^6,7^. However, it remains unknown why such tidal modulation appears to be lacking for intermediate and deep-focus earthquakes.
(v) Diversity in seismicity modulation processes for different objects (e.g. including earthquakes, non-volcanic tremors, volcanic seismicity/tremors, shallow Moonquakes, deep Moonquakes, and Marsquakes) have been linked sporadically in response to diverse stress perturbations from natural harmonic forcing, but it remains enigmatic based on observations and model validations from the viewpoint of the gravity-dependent integrated framework.
(vi) Finally, stress perturbations from natural harmonic forcing on the planetary objects and their satellites predominantly depend on the orientation of the critically stressed faults. However, most of the fault distributions on the planetary objects remain either unidentified, unmapped, or blind at the scales needed for seismicity predictions/modulations of the target of interest^19^.

In this article, we explore an alternative strategy to overcome the above caveats and propose a theoretical modeling approach to test the possibility and diversity of seismicity modulations on planetary bodies and their natural satellites induced by a fault resonance phenomenon, governed by the rate-and-state dependent friction law. In the result and discussion section, we present the diversity in seismicity modulation observed at the Earth, Moon, and Mars, which are only three planetary bodies with data from ground-based monitoring of Earthquakes, Moonquakes, and Marsquakes. Applying our resonance destabilization model to this dataset, we demonstrate the contrast and diversity of induced seismicity modulations on fault interfaces governed by the rate-and-state dependent friction law^33^. Our framework incorporates and elucidates the effect of varying gravitational acceleration “g” which changes drastically for the solar system objects (see Table S1). Finally, a synthetic model prediction for the resonance destabilization process and the influence of the “g-effect” is explored. We validate the theoretical model prediction with contrast/diversity in seismicity modulation on the Earth, Moon, and Mars and summarized the key points of our work in the conclusion section. In the material and methods section, we have presented various seismicity data sets (i.e. Earthquakes, Marsquakes, and Moonquakes) and described the key modeling approach. All supporting figures, tables, relevant datasets, and methods are presented in the supporting documents.

## Results and discussion

### Seismicity modulation on earth

**Figure 1:**
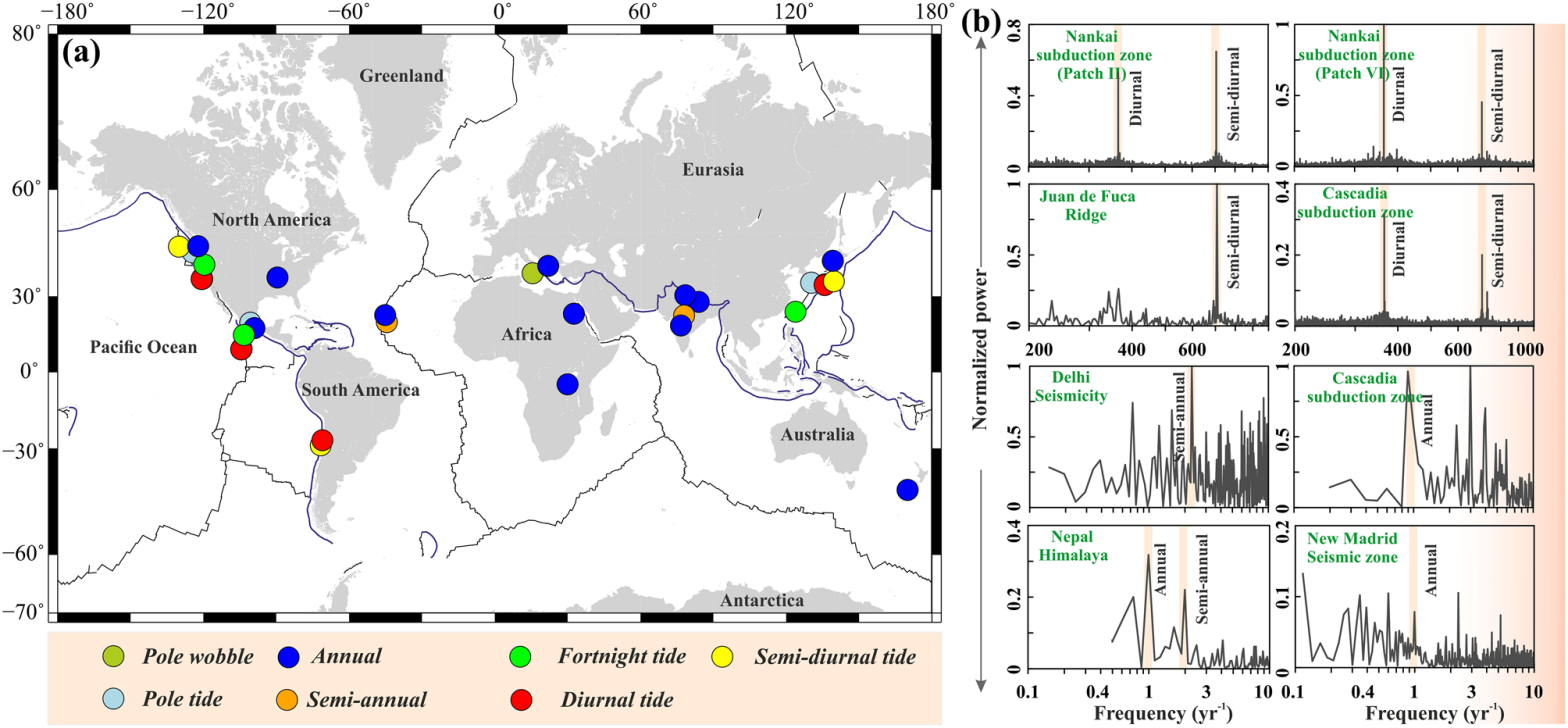
(**a**) Diversity in seismicity modulation observed in the worldwide plate boundary and plate interior domains. Different observed seismicity modulation is represented by the colour circles. (**b**) Power spectrum analysis of some representative seismicity is shown in (**a**). Note that the seismicity associated with the plate boundary regions are exhibited both short-period and long-period seismicity modulation (i.e. Cascadia subduction zone, Nankai subduction zone), whereas the seismicity associated with plate interior regions only exhibits long-period seismicity modulation (i.e. Delhi seismic zone, New Madrid seismic zone).

Spatiotemporal variation of seismicity modulations by the external stress perturbations and destabilization of critically stressed seismogenic fault systems in diverse tectonic settings are well-constrained and monitored on the planet Earth (Fig. 1a). It has been argued that besides tectonic loading, periodic stress variations by tidal loading, seasonal hydrological loading, surface ice/snow loading, reservoir water level fluctuations, glacial isostatic rebound, atmospheric loading, thermos-elastic loading, sediment unloading, seasonal groundwater change, pole tide, pole wobble, etc. are also capable for modulating seismicity^20–27^. From the global diversity in the seismicity modulation, it has been observed that the plate-boundary regions exhibit both short and long-period seismicity modulation (Fig. 1). In contrast, stable plate interior regions are more sensitive to long-period seismicity modulation, and short-period modulation is lacking (Fig. 1).

To demonstrate this, we have considered some representative cases in plate boundary and plate interior regions and analyzed the seismic periodicity, where creep and seismicity bursts correlate with the diverse nature of external stress perturbations (Fig. 1b and supporting documents Figs. S1–S8, Table. S2). Episodes of non-volcanic tremor and accompanying slow slip have been well-monitored in the subduction zones of Nankai from SW-Japan and Cascadia. The power-spectral-density (PSD) clearly shows a burst of tremor activity with periods of 12.4 and 24 to 25 h, identical to the principal lunar and lunisolar tides^13,34^. The non-volcanic tremor events in northern Cascadia also show strong annual peaks (Fig. 1b). This annual modulation is linked with the annual hydrologic cycle-driven downdip shear stress variations on the transition zone beneath Vancouver Island^29^. The geodetic strain has been considered to be linked to the surface load variations. However, in a few cases, the strain variations have also been associated with seasonal seismicity variations^20,25,30,35,36^. This has been well-documented from the mid-crustal micro-seismicity above the base of the seismogenic zone on the Main Himalayan Thrust (MHT), where the PSD plot exhibits both semi-annual and annual peaks, modulated by seasonal continental water storage (Fig. 1b). Further, the micro-seismicity associated with the caldera dynamics of the 2015 axial seamount eruption in the Juan de Fuca ridge shows strong semi-diurnal tidal periodicity in the pre-eruption phase when the fault systems are critically stressed (Fig. 1b), which is modulated by the solid-earth and ocean tide^9,10^.

In contrast to the above examples of relatively faster-moving plate boundaries, the New Madrid seismic zone (NMSZ) in the central USA can be considered one of the well-monitored seismically active stable plate interior regions. The PSD plot of seismicity in the NMSZ exhibits modulation at annual and multi-annual timescales (Fig. 1b). It has been argued that hydrological load-induced stress of the order of a few kPa in the upper Mississippi embayment, is sufficient for seismicity modulation in this seismically active region^28^. Similarly, the low magnitude but moderate seismicity rate linked with the Aravalli Delhi fold belt, on the stable plate-interiors domains of India, exhibits prominent seismicity modulation at a semi-annual seasonal scale due to variation in continental water storage along with long-term groundwater change^37^ (Fig. 1b). Interestingly, tidal modulation is either lacking or not have been reported yet from any stable plate interior regions. It can be claimed that the amplitude of the tidal-induced stress in plate boundary regions is significantly higher compared to the plate interior regions due to the higher ocean tidal loading effect, as most of the plate-boundary regions are situated near the coast. In the Koyna-Warna seismic zone and recently identified Palghar seismic zone of the western coast of India, short-period tidal modulation (i.e. semi-diurnal, diurnal, etc.) should be expected due to the dominance of ocean tidal loading (Figs. S9–11). Surprisingly, in both cases, the short-period tidal modulation is lacking. Rather, the Koyna-Warna seismic zone and the Palghar seismic zone exhibit strong annual and semi-annual periodicity^38,39^, which have been linked to the reservoir water level variation and seasonal rainfall-induced hydrological loading, respectively (Fig. 1b, Figs. S9, 10). These two unique observations confirm that short-period tidal modulation is actually absent in the plate interior regions, even the dominant ocean tidal loading. Although such evidence is rare compared to the dominance of seismicity modulations in the plate-boundary regions, we argue that the lack of sufficient case studies supporting their presence should not be considered evidence for their absence.

### Seismicity modulation on the moon

**Figure 2:**
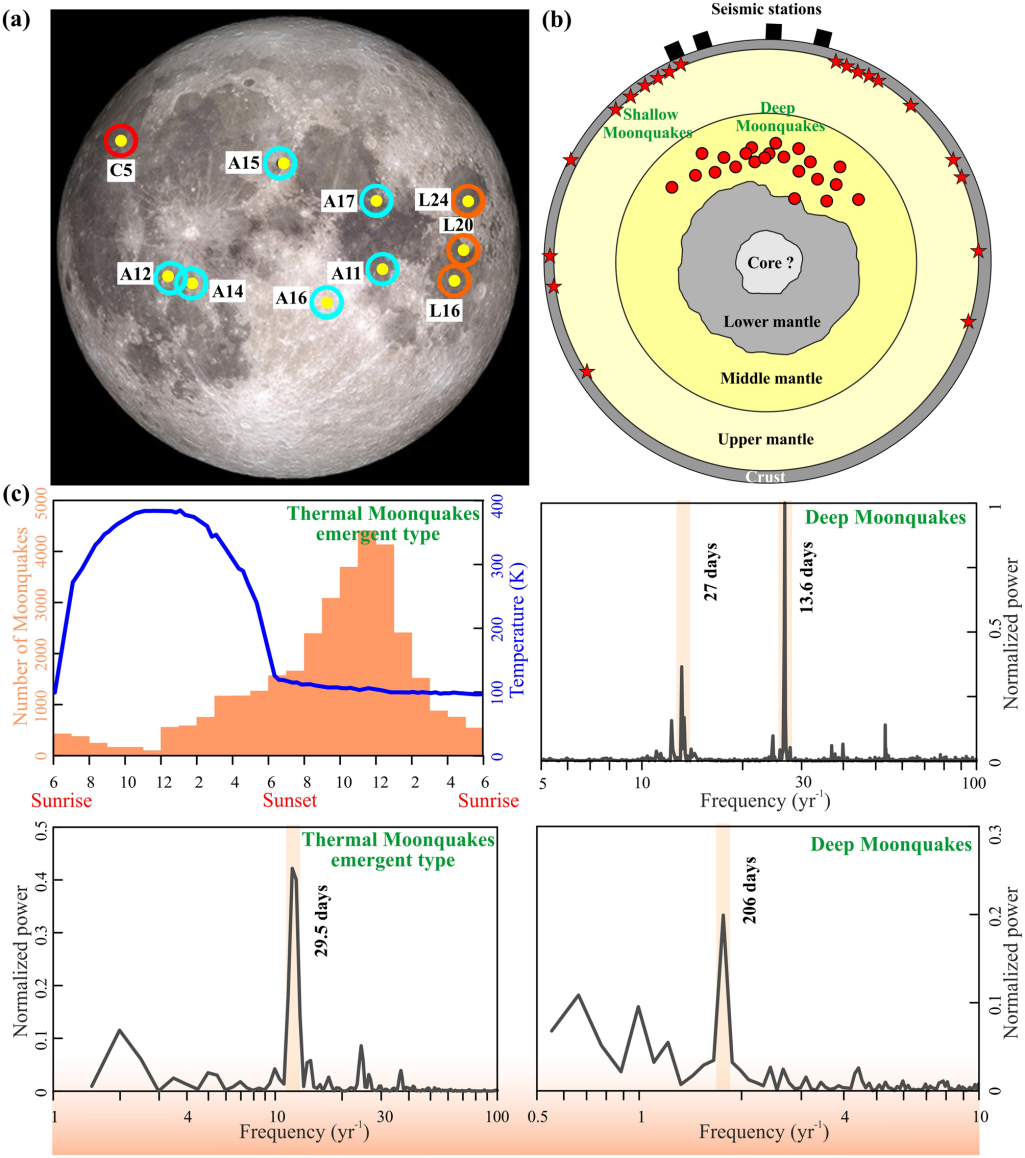
(**a**) Locations of the Apollo mission, Chang’e 5 landing site and Russian Luna sample return sites on the Moon (https://moon.nasa.gov/exploration/moon-missions/). (**b**) Schematic diagram of the interior structure of the Moon. The red stars and circles are represented by the shallow and deep Moonquakes, respectively. Black squares are the network of four seismometers of the Apollo missions (taken from Nakamura, 2020). (**c**) *Left panel top:* Daily histograms of emergent seismic events detected on 8.3 months of Apollo 17 seismic data and Surface temperature variation (blue curve) of Moon surface. *Bottom:* Power spectrum analysis of emergent seismic events. Note that the emergent seismic events exhibit a strong 29.5 days periodicity. *Right panel top and bottom:* Power spectrum analysis of Deep Moonquakes. Note that Deep Moonquakes exhibit significant 27, 13.6, and 206 days periodicity.

The Apollo Lunar seismic experiments (1967–1977) recorded different types of lunar seismic events (Fig. 2a,b), including ~ 2000 deep moonquakes (DMQ) originating at ~ 700–1200 km depth; about 28 shallow moonquakes occurred along young lobate scarps and young thrust faults, nearly 34,000 emergent-type moonquakes, ~ 1700 meteorite impacts and ~ 9 artificial impacts^40^. Meteorite impacts and artificial impacts are clearly of exogenic origin; however other three types are related to endogenic/natural (Table S3). Here, we have considered shallow moonquakes, deep moonquakes, and emergent-type moonquakes for further analysis.

The power-spectral-density analysis of DMQ shows the strongest peak at 13.6 days, followed by a peak around 27 days and 206 days, which have been correlated with the lunar tidal phase (Fig. 2c, Table S3). It has been argued that the DMQ has been related to shear failure induced by mineralogical phase changes^41^ and with the cyclic lunar tidal stress-induced fatigue process^7^. The emergent type moonquakes, recorded by the Lunar Seismic Profiling Experiments at Apollo 17, show strong peaks during the 29.5 days’ lunation period (Fig. 2c), occurring periodically with a sharp peak at sunset time (Fig. 2c, S12, S13). In the daytime maximum lunar surface temperature is reached ~ 360 K at the Apollo 17 landing site and drops to ~ 90 K just before sunrise^42^, hence the diurnal temperature change on the moon’s surface is thought to be a possible triggering mechanism for emergent type moonquakes (Fig. 2c and S12). To confirm this possibility, we analyze the potential triggering of thermal moonquakes by thermo-elastic strain model, adopting the theoretical approach proposed by Berger^43^. Berger^43^ showed that surface temperature variations can induce thermo-elastic strain (and thus stress) variation in a homogeneous elastic half-space (see Sect. 2.3, supporting Material and Methods and Table S4). Constraining the physical properties of lunar regolith/rocks and the diurnal surface-temperature variation, we have computed the daily thermo-elastic stress variation, and it shows a significant amount of stress perturbation of ~ 200 kPa at the depth range of 0–5 km (Fig. S14). This diurnal variation of the thermo-elastic stress change on the lunar regolith is well above the critical triggering threshold of any earthquakes on the planet Earth, hence it is capable of destabilizing the fault system and modulating the seismicity.

Finally, shallow moonquakes are suggested to be of tectonic origin. Although geological structures related to these shallow moonquakes remain controversial, epicenters of eight near-surface shallow moonquakes out of 28 events, have been linked with the nearest fault scarp^8^. It has also been argued that the timing of these events (~ six events) occurred when the Moon was less than 15,000 km from the apogee distance, coinciding with the peak compressional tidal stresses^8^. Nakamura^44^ argued a strong similarity between these shallow Moonquakes and intraplate earthquakes on Earth. Although the different types of lunar seismic events (DMQ and emergent type moonquakes) have been correlated with some periodic stress perturbations, it remains unknown whether convective motion exists in the lunar mantle or it ceased sometime in the geological past. The status of heat exchange mechanism between core to mantle or repeated occurrence of DMQ within ‘nests’ (having dimension ~ 2 km or less) and their tidal sensitivity indicating the existence of fluids or partial melts in the lunar mantle^45,46^ are also unknown.

### Seismicity modulation on mars

**Figure 3:**
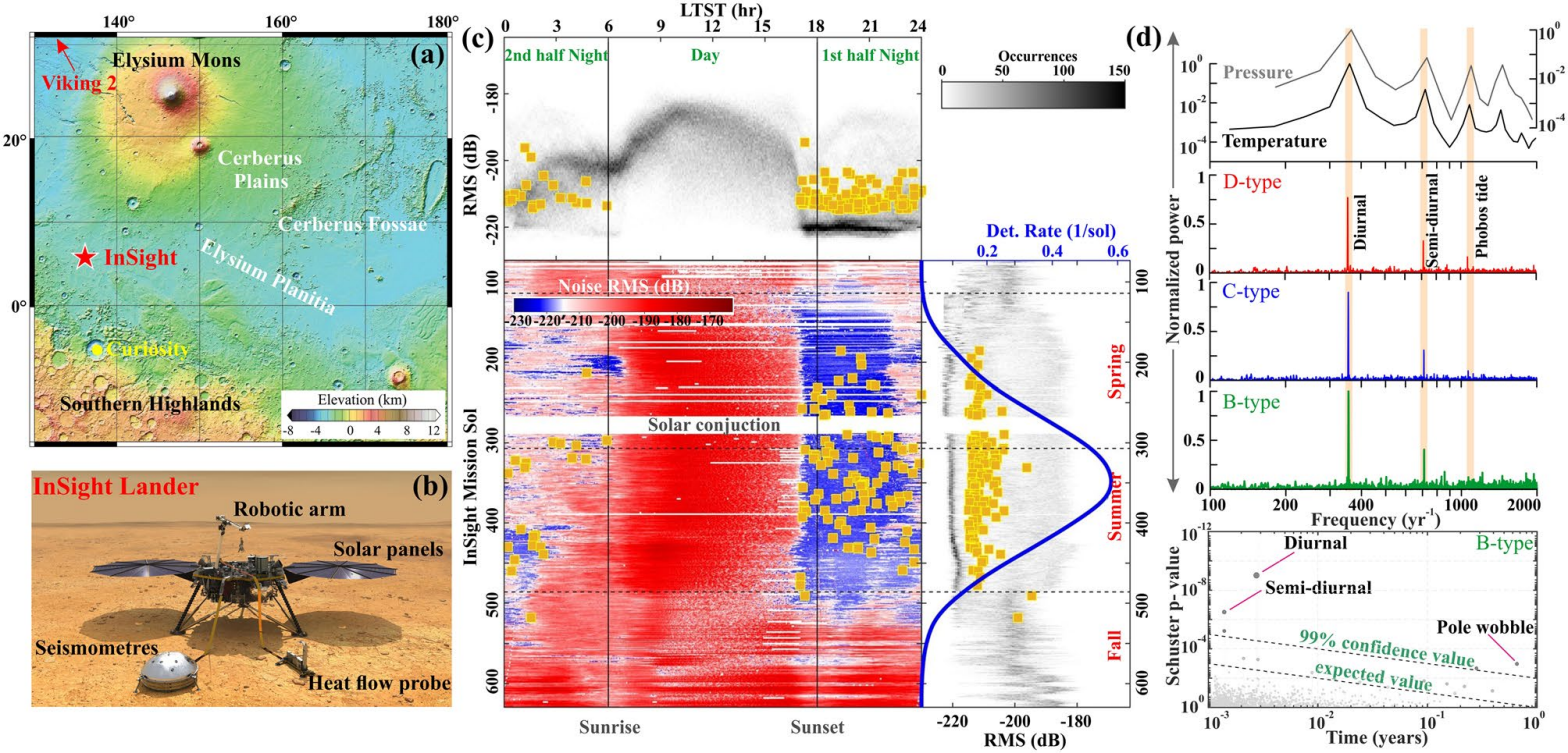
(**a**) Topographic map of the Martian surface. The red star and arrow represent the location of the InSight lander and Viking 2 Lander, respectively (Credit MOLA science team). (**b**) NASA’s InSight spacecraft with its instruments deployed on the Martian surface (https://mars.nasa.gov/insight/). (**c**) *Top panel:* Distribution of noise RMS (2D occurrence count histogram) and event amplitudes (squares) as a function of local time LTST. *Bottom panel:* Noise RMS amplitude (background color) and event detection times (squares) over time. InSight mission Sol is on the vertical, local time LTST on the horizontal axis. Distribution of noise and event amplitudes (squares) as a function of the InSight Mission Sol, dashed horizontal lines indicate the beginning of northern seasons. The blue line represents the kernel density of the detection rate (taken from Knapmeyer et al.^18^). (**d**) *Top panel:* Power spectrum analysis of Martian surface temperature, pressure, and seismic events (i.e. B, C, and D-type seismicity). *Bottom panel:* Schuster spectra analysis for B-type seismicity. Note that the temperature and pressure of Mars and C and D-type Marsquakes exhibit strong diurnal, Semi-diurnal, and Phobos tide, whereas B-type Marsquakes show strong diurnal, Semi-diurnal, and weak pole wobble periodicity.

Based on the surface distribution of faults, thermal contraction, and deployment of a seismometer of the Viking 2 mission, various efforts had been attempted to probe the tectonic activity, the occurrence of marsquakes, and annual moment release in Mars^47–49^. However, none of them provided any robust observations. Hence, to determine the internal structure and the thermal state of Mars, as well as constraining marsquakes, NASA’s InSight mission landed on November 26, 2018^50^, in Elysium Planitia, about 1500 km west of Cerberus Fossae, that was suspected to be seismo-tectonically active region^51^ (Fig. 3a,b). After the landing on Mars, the deployment of the Seismic Experiments for Interior Structure (SEIS) was completed on the 70^th^ Martian day of the InSight mission (i.e. Sol 70) and started recording Marsquakes on Sol 73 (February 9, 2019)^50,52,53^. It has recorded both low-frequency (LF) and ~ 425 high-frequency (HF) events. Further, Clinton et al.^54^ classified the HF family Marsquakes into four types of events (A, B, C, and D-type, respectively) based on the clarity of seismic wave arrivals and the degree of polarization. It has been suggested that A-type is the best quality events (although no HF events are of quality “A”), and D-type events can be considered as artifacts due to wind gusts^55^. Here, we focus on the HF family (~ 425 events) Marsquakes for further study.

From nearly one Martian year of InSight observation, Knapmeyer et al.^18^ have reported that the rate of HF Marsquakes increased after ~ L_S_ = 33° and ceased completely by L_S_ = 187°, following a seasonal variation with a peak seismicity burst during summertime (Fig. 3c), correlated fairly well with the model based on the inclination of the Sun, annual solar tidal forces or annual CO_2_ ice loading. However, the exact mechanism remains poorly understood. Here, we have analyzed the PDS for the marsquakes associated with B, C, and D-type events of the HF family along with the Martian atmospheric pressure and temperature (Fig. 3d). From this analysis, we notice a prominent well-demarcated peak at diurnal and semi-diurnal period for all three B-, C- and D-type of HF family. In addition, the C- and D-type events (events that might be artifacts, e.g. wind gusts) also show relatively weak peaks at 7.36 h Phobos’ tidal periods, and such periodicity is also reflected in the atmospheric pressure and temperature variation (Fig. 3d). However, in the B-type of events (the best quality of Marsquakes), we have not noticed any periodicity related to Phobos’ orbit, as claimed in an earlier study connected to groundwater pore-pressure effects from the cooling of the Martian surface^17^. Hence, we suggest that the peak at Phobos’ tidal period that occurred in the C- and D-type events are possibly correlated with the environmental non-seismic noise. To crosscheck this observation further, we have computed spectra of Schuster p-values for the B-type events (Fig. 3d below panel), and it exhibits statistically significant diurnal and semi-diurnal periodicity but no periodicity related to Phobos’ orbit. We have also noticed another prominent periodicity close to ~ 238 days, which appears to be fairly close enough to the reported Chandler Wobble of Mars (~ 206.9 days) (Fig. 3d below panel). Moreover, we have also analysed the periodicity of the Marsquakes, considering the full InSight dataset (1200 days) and the results are consistent (Fig. S15).

**Figure 4:**
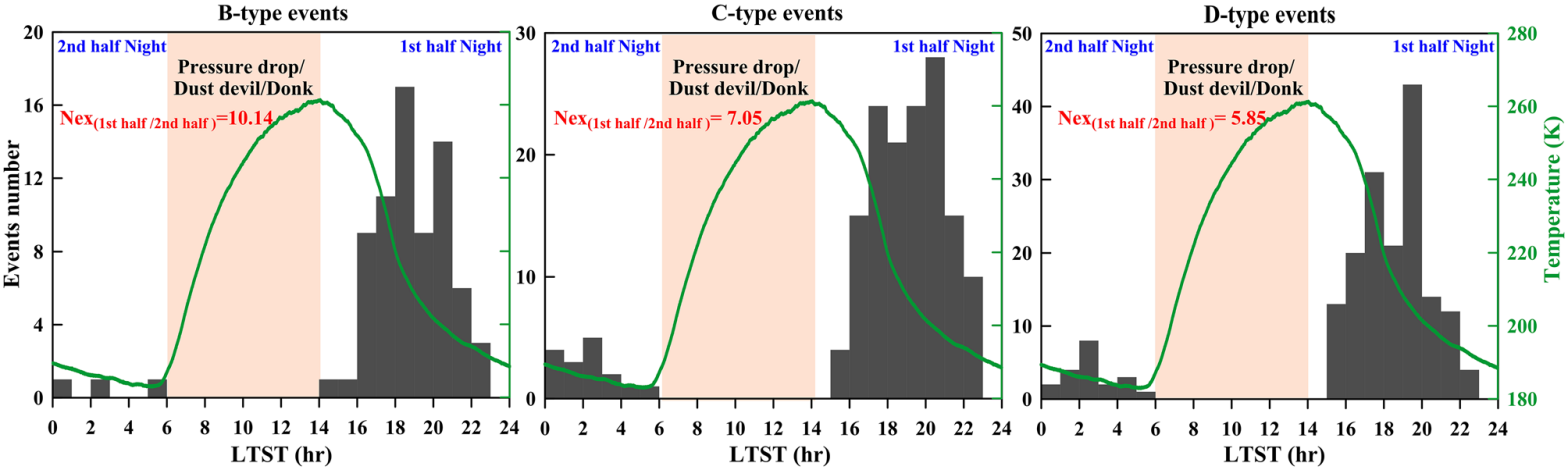
Hourly histogram of the B, C and D-type HF-Marsquakes. The green curve represents the diurnal variation of the temperature on the Martian surface.

Furthermore, it has also been argued that the semi-diurnal periodicity in the B-, C- and D-type events might be partly due to tidal effects or contributions from the different detection probabilities during the first half (17 to 24 LMST) and the second half (24 to 6 LMST) of the nights (Fig. 4). However, the strong diurnal periodicity is certainly due to different detection probabilities. We have not considered any HF events from the daytime (6–17 LMST) due to strong sources from dust devil, donk, and other artificial sources of non-seismic noise (Fig. 4). The diurnal variation in atmospheric pressure and temperature along with the comparison of P–T stability phase of water (or ice) and carbon dioxide (or dry ice) (Fig. S16), clearly indicates no such mechanical role and associated pore-pressure build-up in the aquifers confined below a cryosphere as Mars cools between the first and second half of the nights. In fact, during the second half of the night, Mars cools significantly compared to the first half of the night. Hence, we expect much more pore-pressure build-up in the aquifers confined below a cryosphere, and that should lead to the dominance of marsquakes. However, the consequence is just the reverse (Fig. 4). From these observations, we argue that diurnal and semi-diurnal periodicity in the HF family appears to be an artifact due to the different detection probabilities. Moreover, the variation in noise distribution between the first half and second half of the night is also in better agreement with this argument (Fig. 3d). Hence, we summarized that seasonal variation with an annual seismicity burst, and seismic periodicity at polar wobble periods in the marsquakes appears to be a natural signature. However, the diurnal and semi-diurnal periodicity, along with Phobos’ tidal period, are possible artifacts.

### Resonance destabilization model and theoretical prediction

The resonance destabilization model has been well-studied theoretically^56–58^, at laboratory scales^59^ and via natural observations^36,60^. It has been proposed that the fault system may change from a stable domain to a stick–slip domain due to the slight variation of the periodic external stress and modulated seismicity. The velocity (V) response of a fault system due to variation of external stress having period T and amplitude of shear stress τ_1_ and normal stress σ_1,_ which is expressed as^32^:

$$V={V}_{L}+Im\left[{V}_{1}exp\left(i\omega t\right)\right],$$

where ${V}_{1}=\rho v {\text{exp}}(-i\gamma v)$, ${V}_{L}$ is the plate or long-term velocity, $Im$ is the imaginary part, and $\omega =\frac{2\pi }{T}$ (for more detail see “Material and Methods” section). To test such possibility and diversity in seismicity modulations on the Planetary bodies and their natural satellites, we explore the resonance destabilization process under a rate-and-state friction framework^33^, with a critical emphasis on gravitational acceleration “g” of different solar system objects.

**Figure 5:**
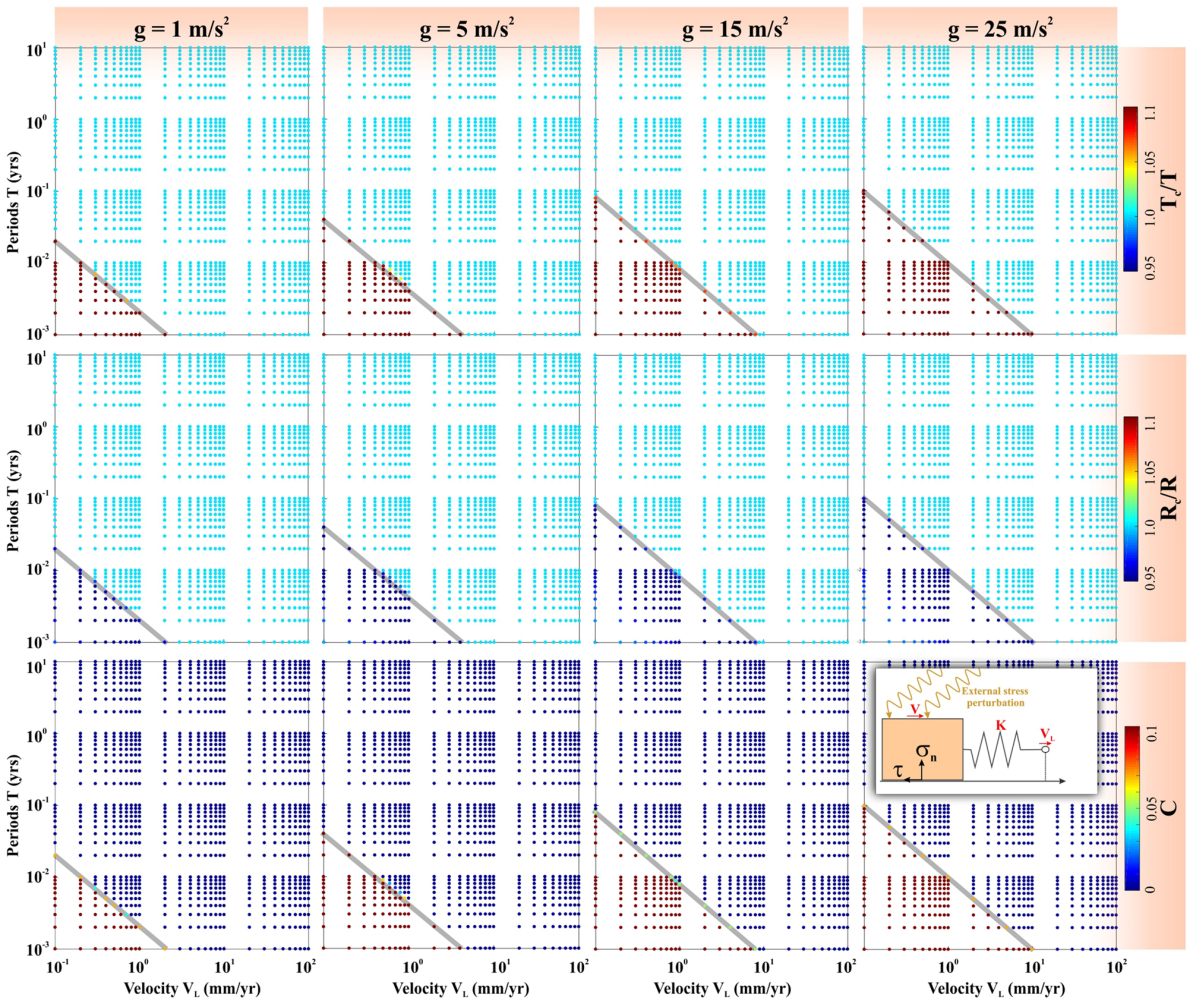
Variation of the Fault resonance parameters that are estimated from the resonance destabilization process under rate-and-state dependent friction. The fault resonance parameters $\frac{{T}_{c}}{T}$, $\frac{{R}_{c}}{R}$ and cost function C are estimated from the resonance destabilization process by varying periods of external stress perturbation (T) and velocity of the faults (V_L_), considering the length of the modulating fault patch or dimension of the slipping zone (R) as 50 km, depth of the seismicity (Z) as 35 km and gravitational acceleration (g) 1, 5 15 and 25 m/s^2^ respectively. The grey line demarcated the possible resonance destabilization zone to the not possible resonance destabilization zone. Note that resonance destabilization occurs, when the critical period of external stress perturbation (T_c_) is nearly equal to the period of external stress perturbation (T). The critical dimension of the slipping zone (R_c_) is very close to the dimension of the slipping zone (R) and cost function C should be close to zero.

In Fig. 5, we present the spatial stability field of naturally occurring seismicity modulations induced by fault destabilization mechanism due to a resonance phenomenon. To characterize the external stress perturbations into resonance destabilization models, it must satisfy the following three criteria. The forcing period (T) must be close to the critical period of forcing (T_c_), the length of the resonating patch (R) must be close to the critical length of the resonating patch (R_c_), and finally, the cost function (C) should be close to zero. Therefore, in the V_L_ vs. T plot, resonance destabilization domains are characterized by $\frac{{T}_{c}}{T} \to 1$, $\frac{{R}_{c}}{R} \to 1$, and C → 0. We have demarcated the spatial domain of the resonance patch (where we expect natural seismicity modulation) to the non-resonance patch (where seismicity modulation can be considered as an artifact) by a critical boundary in the V_L_ vs. T plots (marked by gray lines in Fig. 5). We have varied V_L_, T, gravitational acceleration “g” systematically, over the natural ranges of these physical parameters. We have varied the plate velocity (V_L_) from 10^–1^ to 10^2^ mm/yr, which covers the overall plate motion from plate boundary to plate interior region in the entire globe. We varied the forcing period of modulation (T) from 10^–3^ to 10^1^ years, including diverse types of the periodic stress perturbations process that are responsible for the diverse nature of seismicity modulation on the solar system objects (as we discussed in the previous sections).

**Figure 6:**
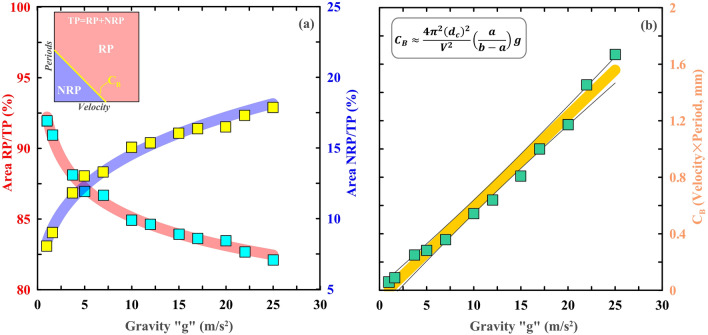
*Left panel:* Variation of the ratio of the resonant patch (RP) and non-resonant patch (NRP) as a function of gravity (g). Inset Figure shows a schematic representation of a resonant patch (RP) and a non-resonant patch (NRP). Yellow line (marked as C_B_, critical boundary) that has demarcated RP from NRP. *Right panel:* Variation of the C_B_ as a function of the gravity (g). Note that C_B_ can be expressed as the function of gravity and the rate and-state frictional parameters (**a**,**b**), and the critical slip distance d_c_.

Under such assumptions, it has been observed that with a progressive increase in gravitational acceleration “g” of solar system objects, the effective area of the resonance domain gradually decreased with an increase of the non-resonance domain in each V_L_ vs. T spatial plots (Fig. 5). More specifically, it has been noted that there is ~ 17% increase of non-resonance patch with respect to the total area in V_L_ vs. T spatial domain with an increase in gravitational acceleration “g” from 1 to 25 m/s^2^ (Fig. 6). Further, from this gravitational acceleration-dependent theoretical model prediction, we find that the critical boundary (C_B_) demarcating the boundary between the non-resonance patch and the resonance patch in the V_L_ vs. T spatial domain, linearly relates to gravitational acceleration “g” and frictional parameters (e.g. critical slip distance ${d}_{c}$ and frictional parameters a and b). We suggest that in our resonance destabilization theoretical model, d_c_ varies in the range of 10^−6^ to 10^−3^ m, which is consistent with the laboratory-estimated value of this parameter^61^.

### Model prediction and diversity in seismicity modulation

**Figure 7:**
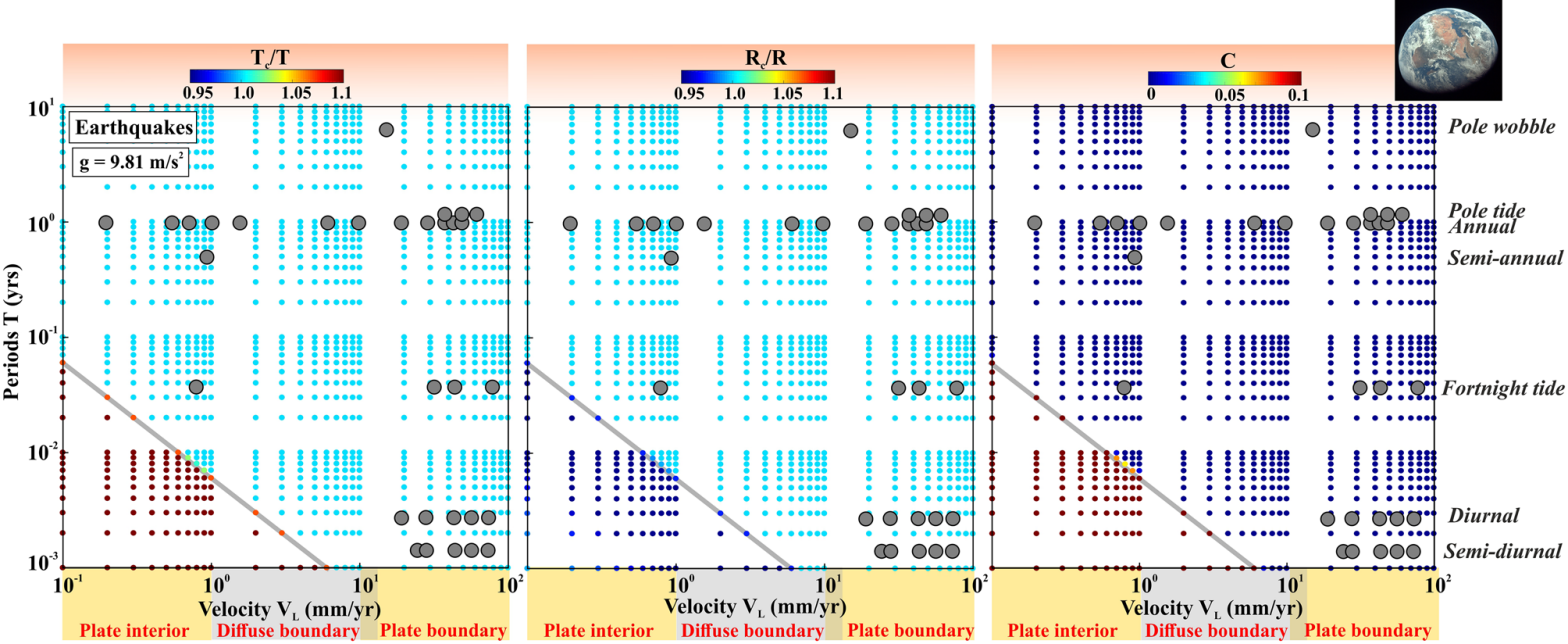
Variation of the Fault resonance parameters that are estimated from the resonance destabilization process under rate-and-state dependent friction. The fault resonance parameters $\frac{{T}_{c}}{T}$, $\frac{{R}_{c}}{R}$ and cost function C are estimated from the resonance destabilization process by varying periods of external stress perturbation (T) and velocity of the faults (V_L_), considering the length of the modulating fault patch or dimension of the slipping zone (R) as 50 km, depth of the seismicity (Z) as 35 km and gravitational acceleration (g) of Earth (9.81 m/s^2^). Diversity in seismicity modulation observed in the worldwide plate boundary and plate interior domains are marked by the grey dots. Note that the short-period modulation in the plate interior region is absent.

**Figure 8:**
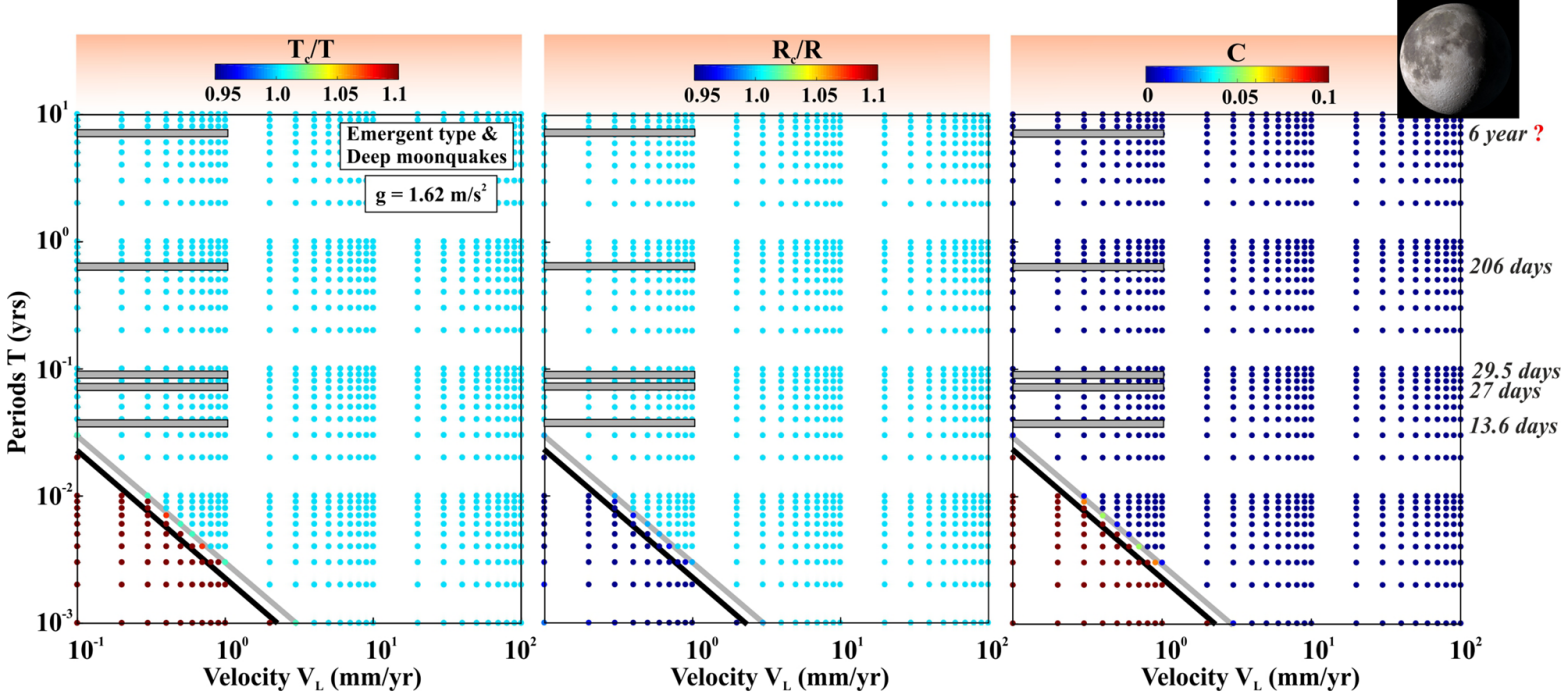
Variation of the Fault resonance parameters that are estimated from the resonance destabilization process under rate-and-state dependent friction. The fault resonance parameters $\frac{{T}_{c}}{T}$, $\frac{{R}_{c}}{R}$ and cost function C are estimated from the resonance destabilization process by varying periods of external stress perturbation (T) and velocity of the faults (V_L_), considering the length of the modulating fault patch or dimension of the slipping zone (R) as 50 km, depth of the seismicity (Z) as 35 for emergent type moonquakes and (R) as 2 km (i.e. equivalent dimension of the “nests”), depth of the seismicity (Z) 1000 km for deep Moonquakes (DMQ) respectively along with gravitational acceleration (g) of Moon as 1.62 m/s^2^. The observed seismic periodicity on moonquakes are marked by the horizontal grey bars. The black and grey inclined lines represent the resonant to the non-resonant area for emergent type and deep moonquakes, respectively.

**Figure 9:**
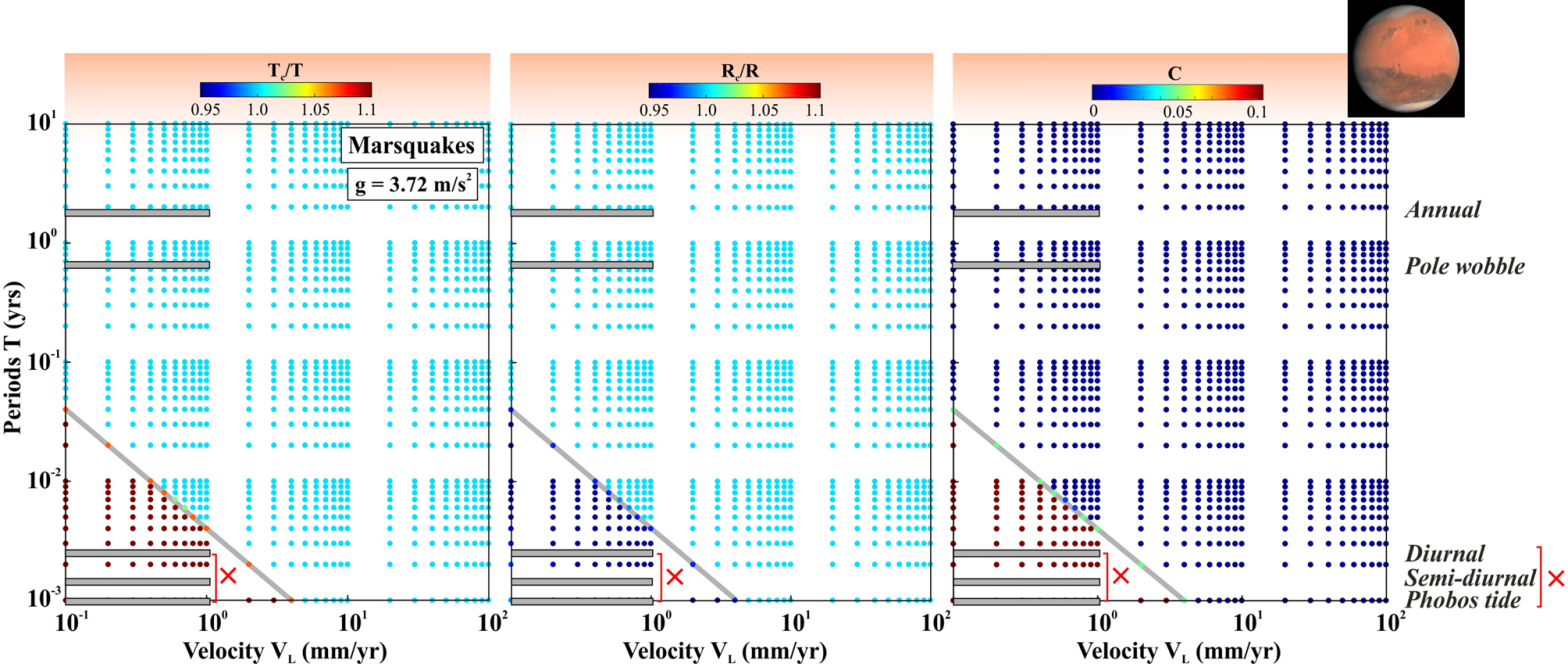
Variation of the Fault resonance parameters that are estimated from the resonance destabilization process under rate-and-state dependent friction. The fault resonance parameters $\frac{{T}_{c}}{T}$, $\frac{{R}_{c}}{R}$ and cost function C are estimated from the resonance destabilization process by varying periods of external stress perturbation (T) and velocity of the faults (V_L_), considering the length of the modulating fault patch or dimension of the slipping zone (R) as 50 km, depth of the seismicity (Z) as 35 km and gravitational acceleration (g) of Mars (3.72 m/s^2^). The observed seismic periodicity on marsquakes are marked by the grey bars.

To test the robustness of model prediction of resonant destabilization process, influenced by gravitational acceleration “g” in response to diversity in seismicity modulation for the earthquakes, moonquakes, and marsquakes, we have projected them over V_L_ vs. T spatial domain, respectively (Figs. 7, 8 and 9). From the associated creep and seismicity bursts on a typical Earth-like planet, it is observed that the relatively short-period modulation, including semi-diurnal, diurnal, and fortnight tidal constituents, appears to be less common in stable plate interior and diffuse deformation boundaries, and the long-period modulations including semi-annual, annual, pole tide, pole wobble, multi-annual time scales appear to be more common. However, the plate boundary regions are equally susceptible to both short-period and long-period seismicity modulation (Fig. 7). This can further strengthen the claim that the short-period tidal modulation is absent in the plate interior regions.

In the case of moonquakes (Fig. 8), as gravitational acceleration “g” is substantially lower than the Earth, all types of observed seismicity modulations, including emergent type moonquakes and DMQ are possible and can be considered as natural. Because of the extremely low gravitational acceleration of the Moon (g = 1.62 m/s^2^), repeated occurrence of DMQ within ‘nests’ and their tidal rhythms (i.e. 13.6 days and 27 days’ periodicity) are possible, even though Moon behaves as a plate interior region of the Earth. Moreover, our resonance destabilization model, lack of the heat-flow anomaly on the lunar surface, and presence of tidal modulation for the DMQ also indicate the deformation rate is ~ 0.1 mm/yr (i.e. comparable with NMSZ in the central United States^62^) at the depth range of 700–1200 km.

Finally, the seismicity modulation related to marsquakes also indicates better agreement between the theoretical model prediction and natural observations. From Fig. 9, it has been observed that annual seismicity burst and seismic periodicity at polar wobble periods appear to be a natural consequence as it lies in the domain of resonance patch. However, diurnal and semi-diurnal periodicities, along with Phobos’ tidal period, lie in the domain of the non-resonance patch, and hence clearly indicate an artifact. In fact, the absence of short-period seismicity modulation in the HF family (B-type events) also indicates that the plate tectonics process and rigorous nature of mantle convection possibly have ceased at the present day. The lack of mantle convection and hence the absence of effective heat exchange with the core possibly indicates that Martian core convection has terminated. The clear proxy from the Martian rock magnetization indicates that the magnetic field once existed on Mars, and it does not exist in recent times^63,64^. Although, newly discovered repetitive low-frequency events have been related to magma movements linked with volcanic activity in the upper mantle beneath the Cerberus Fossae^65^. It remains enigmatic whether such repetitive occurrence of low-frequency events is really a conclusive proxy for Martian’s subcrustal activity, substantially higher than anticipated, or it is related to the non-seismic noise of the Martian environments.

**Figure 10:**
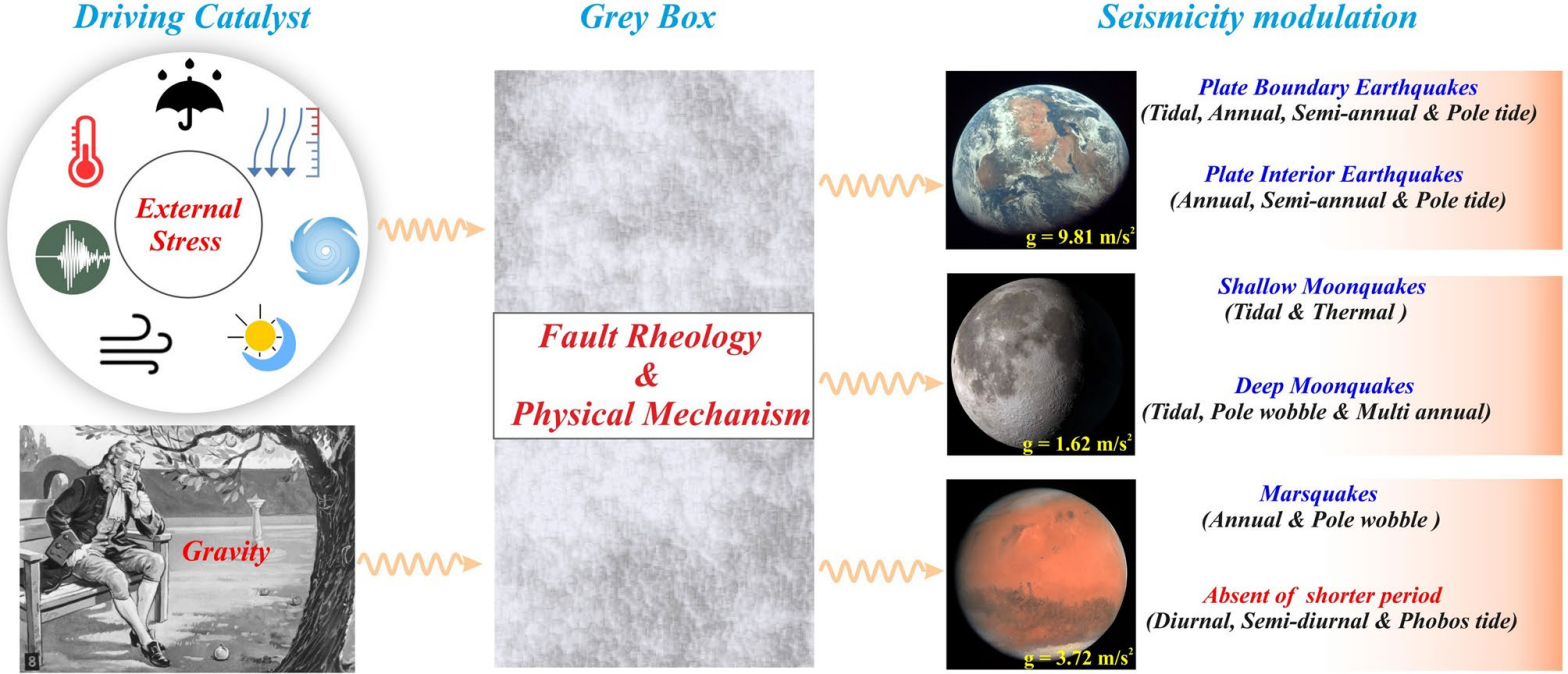
Diversity and contrast of seismicity modulation for planetary seismology behave like a grey box analog. Variations in gravitation accelerations “g”, along with diverse types of natural external harmonic processes, are predominantly responsible for seismicity modulation on the planetary bodies and their natural satellites. The various type of external periodic forcing is shown in the circles in the clockwise direction as rainfall, pressure, typhoons, tides, wind, seismic wave generated from the distant earthquake, and temperature, respectively.

The exact strength and state-of-stress of the seismogenic faults, including fault rheology and orientation of the critically stressed faults in the planetary bodies, exoplanets and their satellites, are largely unknown, and hence understanding the physics of the seismicity modulation for planetary seismology behaves like a grey box analog (Fig. 10). Using constraints from natural observation and theoretical modeling perspectives, we hypothesize that the variations in gravitational acceleration “g” of different solar system objects combined with diverse types of natural external harmonic processes are predominantly responsible for seismicity modulation on the planetary bodies and their natural satellites. Gravity-induced resonance destabilization model appears to be in better agreement with the contrast/diversity in seismicity modulation on the Earth, Moon, and Mars. We acknowledge that the presence of anomalous crustal fluid^66–68^, variation in frictional parameters^69,70^, critical triggering thresholds^71–73^, fault geometry and rheology^69,74^, fault gauge accumulation^70^, etc., can make the seismic triggering/modulation phenomenon extremely complex and non-linear. Nevertheless, our robust observations and gravity-induced resonant destabilization model clearly demonstrate diversity in seismicity modulations of the solar system objects in an integrated approach. We are optimistic that the present hypotheses can be tested further with the availability of more seismological observations from different planetary bodies, exoplanets and their moons in future missions of space exploration.

## Conclusions

From the above study based on the resonance destabilization model under rate-and-state dependent frictional law and comparison with seismicity modulation in Earth, Moon, and Mars, we have summarized the following salient points:

1. We observe that the plate boundary region in the Earth shows both short-period (diurnal, semi-diurnal, etc.) and long-period (annual, semi-annual, pole tide, etc.) modulation, whereas the plate interior region exhibits only long-period modulation.
2. The annual and polar wobble periodicity observed in the high-frequency marsquakes indicates a natural origin, whereas diurnal and semi-diurnal periodicity, along with Phobos’ tidal period, are likely artifacts due to different detection probabilities and non-seismic noise.
3. The deep moonquakes are susceptible to both lunar tidal phase (13.6 days and 27 days) and long-period pole wobble (206 days) modulation, whereas shallow emergent-type moonquakes show a seismic periodicity at the lunation period (29.5 days).
4. From the rate-and-state friction-dependent resonance destabilization model, it has been observed that the effective area of the non-resonance domain increases with increasing gravitational acceleration. Hence, we suggest that the gravity-induced resonance destabilization model appears to be in better agreement with the diversity in seismicity modulation on the Earth, Moon, and Mars. Finally, we have suggested that the resonance destabilization model presented here can produce comprehensive results linked to seismicity modulation by different exogenous processes in planetary bodies and their natural satellites.

## Material and methods

### Earthquakes/tremors, moonquakes, and marsquakes datasets

In order to analyze the seismic periodicity, we have considered several seismicity/tremor catalogs from Earth, Mars, and Moon. In the case of Earth, we have considered some selective and well-established seismicity data from the New Madrid seismic zone (http://www.memphis.edu/ceri/seismic/catalog.php), South West Japan (http://www-solid.eps.s.u-tokyo.ac.jp/~sloweq/), Cascadia subduction zone (https://pnsn.org/tremor), Delhi-Haridwar region (https://seismo.gov.in/), Nepal Himalaya (http://www.seismonepal.gov.np/) and Juan De Fuca region (http://ddrt.ldeo.columbia.edu/Axial, Ref.^75^). For the seismicity modulation of Moon, we have considered emergent type Moonquakes and deep moonquakes catalogs, recorded by a small network of four geophones landed on the lunar surface during the Lunar Seismic Profiling Experiment (LSPE) for the period of August 1976 and April 1977 (i.e. it detected ~ 50,000 moonquakes, archived at https://data.mendeley.com/datasets/g3yccthhwn/2, Ref.^76^. Further, the deep Moonquakes recorded by Apollo Lunar Surface Experiment (ALSEP) stations from 1969 to 1977, which is monitored by the Galveston Geophysics Laboratory of the University of Texas and archived from http://www-udc.ig.utexas.edu/external/yosio/PSE/catsrepts/. Finally, to characterize the modulation in the marsquakes, we have exploited the catalog from the period of 12 January, 2019 and 31 August, recorded by the NASA InSight mission (Interior exploration using Seismic Investigations, Geodesy, and Heat Transport). It can be accessed at https://ars.els-cdn.com/content/image/1-s2.0-S0031920120302739-mmc2.pdf, Refs.^18,54^.

### Seismic periodicity analysis

To estimate the periodicity of seismicity associated with the Earth, Moon, and Mars, we have adopted the spectra of Schuster p-values proposed by Ader and Avouac^77^ and Power Spectra density analysis. The spectra of Schuster p-value is expressed as:

1 $$p={e}^{-{D}^{2}/N},$$

where $p$ is the Schuster probability value, $N$ is the number of events in the seismicity catalog and $D$ is the time span between the start and end point of the seismicity catalog^77^. Further, for Power Spectra density analysis, we have generated continuous seismicity time series by converting the entire period of the earthquake time series into the number of events per hour and estimated periodicity by Power Spectra density analysis.

### Thermoelastic stress computation on the Moon’s surface

To find out the correlation between surface-temperature induced thermo-elastic stress and associated deformation on the Moon's surface, we have adopted the theoretical approach proposed by Berger^43^. It has been proposed that in a homogeneous elastic half-space, the surface temperature variation induced thermo-elastic strain variation can be expressed as^43^:

2 $$T\left(y=0\right)={T}_{0}{e}^{i(\omega t+kx)},$$

where x represents horizontal position, y represents depth and t represents time, ${T}_{0}$ is the amplitude of periodic temperature variation, $\omega$ is the angular frequency, and k is the horizontal wave number. Further, different components of thermo-elastic stress are expressed as:

3 $${\sigma }_{xx}=\left(\lambda +2G\right){e}_{xx}+\lambda {e}_{yy}+\lambda {e}_{zz},$$

4 $${\sigma }_{yy}=\lambda {e}_{xx}+\left(\lambda +2G\right){e}_{yy}+\lambda {e}_{zz},$$

5 $${\sigma }_{xy}=2G{e}_{xy},$$

where $G$ is the shear modulus denoted by $G=E/2(1+\nu )$, $\lambda$ is the Lame’s parameter that is represented by $\lambda =E\nu /(1+\nu )(1-2\nu$, ν is the Poisson’s ratio and E is Young’s modulus. For 2-D stress computation, we have assumed ${e}_{zz}$=0 (For detail, see supporting documents).The other physical parameters and given in Table S4.

### Tidal stress computation model

The SPOTL program is used to compute stress exerted by tidal loading^2,3^. The tidal strains are calculated from the corresponding positions of the Sun and Moon by assuming Green’s functions from Guttenberg-Bullen Earth model^78^, considering an elastic and spherical Earth model with the satellite estimated Cartwright-Tayler constituent amplitudes with 2nd-degree Love numbers as h = 0.6114, k = 0.3040, and l = 0.0832. During the estimation of tidal strain, different ocean tidal models were used as the GOT4.7 global oceans tidal model^79^ and the regional ocean tidal model produced by the Oregon State University (OSU) for the United States western coast^80^. It combined the loading from both models and used eight major short-period tidal constituents (K1, K2, M2, N2, O1, P1, Q1, and S2) to calculate the strains at the specific point accurately. Finally, to compute the tidal stress from the estimated strains, we have considered an Elastic modulus of 30 GPa and a Poisson ratio of 0.25. The strains are rotated by using linear-elastic constitutive equations on the azimuth of the fault plane and estimate the fault normal stress and fault parallel shear stress. The Coulomb failure stress is calculated considering the frictional coefficient as 0.3.

### Resonance destabilization model

The critical stressed fault systems are very sensitive to periodic external stress perturbation^32,56,58^. It has been proposed that a small variation in external stress perturbation on the fault system is capable of destabilizing the fault system. To know the effect of external stress perturbations on the fault system, we estimate the velocity (V) response of a fault system due to variation of external stress having period T and amplitude of shear stress τ_1_ and normal stress σ_1,_ which is expressed as^32,81^:

6 $$V={V}_{L}+Im\left[{V}_{1}exp\left(i\omega t\right)\right],$$

where ${V}_{1}=\rho v {\text{exp}}(-i\gamma v)$, ${V}_{L}$ is the plate or long-term velocity, $Im$ is the imaginary part and $\omega =\frac{2\pi }{T}$.

The parameters $\rho v$ and $\gamma v$ are given by:

7 $$\rho v=\frac{{V}_{L}q{\tau }_{ss}}{k{d}_{c}}\sqrt{\frac{{\left\{q\left({\epsilon }_{\sigma }\left(1-\frac{\alpha }{{\mu }_{ss}}\right)-{\epsilon }_{\tau }\right)\right\}}^{2}+{\left({\epsilon }_{\sigma }-{\epsilon }_{\tau }\right)}^{2}}{{\left(1-\frac{{{k}_{c}\left(\frac{q}{{q}_{c}}\right)}^{2}}{k}\right)}^{2}+{\left(q\left(1-\frac{{k}_{c}}{k}\right)\right)}^{2}}},$$

8 $$tan \left(\gamma v\right)=\frac{{q}^{2}\left(1-\frac{{k}_{c}}{k}\right)\left[\left({\epsilon }_{\sigma }\left(1-\frac{\alpha }{{\mu }_{ss}}\right)-{\epsilon }_{\tau }\right)\right]-\left({\epsilon }_{\sigma }-{\epsilon }_{\tau }\right)\left(1-\frac{{{k}_{c}\left(\frac{q}{{q}_{c}}\right)}^{2}}{k}\right)}{q\left[\left({\epsilon }_{\sigma }\left(1-\frac{\alpha }{{\mu }_{ss}}\right)-{\epsilon }_{\tau }\right)\right]\left(1-\frac{{\left(\frac{q}{{q}_{c}}\right)}^{2}}{\frac{k}{{k}_{c}}}\right)-q\left(1-\frac{1}{\frac{k}{{k}_{c}}}\right)\left({\epsilon }_{\sigma }-{\epsilon }_{\tau }\right)},$$

where

9 $$\left.\begin{array}{c}q=\frac{{V}_{L}}{{d}_{c}}\frac{2\pi }{T}\\ {\epsilon }_{\sigma }=\frac{{\sigma }_{1}}{{\sigma }_{*}}\\ {\epsilon }_{\tau }=\frac{{\tau }_{1}}{{\mu }_{ss}{\sigma }_{*}}\\ {\mu }_{ss}={\mu }_{*}+\left(a-b\right)log\left(\frac{{V}_{L}}{{V}_{*}}\right)\\ {k}_{c}=\frac{\left(b-a\right){\sigma }_{*}}{{d}_{c}} \\ {q}_{c}=\sqrt{\frac{b-a}{a}}\\ {T}_{c}=2\pi \sqrt{\frac{a}{b-a}}\frac{{d}_{c}}{{V}_{L}}\end{array}\right\}.$$

Here ${T}_{c}$ is the critical period of excitation, a and b are rate- state-dependent frictional parameters, ${d}_{c}$ is the characteristic slip distance,${\mu }_{ss}$= steady-state frictional coefficient, $q$ is the dimensionless frequency, ${k}_{c}$= critical stiffness of the fault patch.

The T, V_L_, σ_1_, τ_1_ and k. parameters are required for applying the model in to real natural cases. Out of them, V_L_ and T of a fault system is known. The amplitude of σ_1_ and τ_1_ on the fault plane can be calculated accurately. The stiffness (k) of a fault patch is related to the length of the fault patch (R) and shear modulus, which is expressed as:

10 $$k=\frac{\gamma G}{R}.$$

Here $\gamma =\frac{7\pi }{16}$ for circular cracks^82^.

Further, it is important to note that in rate and state friction, the parameters a and b cannot be estimate independently, as they are always associated with normal stress. The parameter $\alpha$ can be neglected ($\alpha$ = 0) or considered as $\alpha =\frac{{\mu }_{ss}}{3}$ in rate-and-state friction as proposed by Perfettini and Molinari^83^. Hence, a strong resonance amplification is only possible, when the denominator of Eq. (7) is very close to zero, which is independent of $\alpha$. Hence, the $\alpha$ is not so significant when discussing the conditions for a strong resonance amplification. As a result, now the model only depends on the $\epsilon =\frac{b-a}{a}$, $A=a{\sigma }_{* }=a\rho gz$, ${d}_{c}$, ${\epsilon }_{\sigma }$ and ${\epsilon }_{\tau }$ .

By replacing $\epsilon =\frac{b-a}{a}$, in the equation ${T}_{c}=2\pi \sqrt{\frac{a}{b-a}}\frac{{d}_{c}}{{V}_{L}}$ and $A=a{\sigma }_{* }=a\rho gz$ and ${k}_{c}=\frac{\left(b-a\right){\sigma }_{*}}{{d}_{c}}$ in Eq. (10), the ${T}_{c}$ and ${R}_{c}$ can be expressed as following:

11 $$\left.\begin{array}{c}{T}_{c}\approx \frac{2\pi }{\sqrt{\epsilon }}\frac{{d}_{c}}{{V}_{L}}\\ {R}_{c}\approx \frac{\gamma G}{A\varepsilon }{d}_{c}\end{array}\right\},$$

where *R*_*c*_ is nucleation size/ critical slipping patch of fault^84^.

When the model is applied to natural cases, three unknown parameters (i.e. ε, d_c_, and A) have to be inferred. To characterize the resonance process, the parameters ε, d_c_, and A are inverted and constrained by the two parameters T and R (i.e. forcing period and the length of the resonating patch). Further, we have considered lithostatic pressure as σ_litho_ = ρgz where ρ = 3000 kg/m^3^ is the rock density taken, g is the gravitational acceleration, and z is the mean depth of the resonating patch. The other constants of the model are $\gamma =\frac{7\pi }{16}$ and G assumes as 30 GPa. The parameter A spans a large range of admissible values, varying from 10^–9^ σ_litho_ to σ_litho_. For the individual value of A, the parameters ε and d_c_ are estimated by minimizing the cost function, which is given as:

12 $$C=\sqrt{\frac{{\left(1-\frac{T}{{T}_{c}}\right)}^{2}+{\left(1-\frac{R}{{R}_{c}}\right)}^{2}}{2}.}$$

From the above equation, it has been observed that the fault resonance model depends upon several parameters. The amplitude of velocity perturbations is the function of long-term velocity, characteristic slip distance, fictional parameters a and b, gravitational acceleration, critical periods of external forces and the critical slipping patch of fault. Further, the cost function is minimized using matlab’s routine fminsearchbnd with d_c_ varying from 10^−6^ to 0.1 m and ε from 10^−5^ to 10, respectively. We have also varied the gravitational acceleration g from 1 to 25 m/s^2^. Finally, we apply this resonance destabilization model to address the diversity of seismicity modulation in the Earth, Moon and Mars in response to periodic external stress perturbations.

### Supplementary Information

Supplementary Information.

## Data Availability

All the datasets used in the present study are openly available in the public domain and mentioned in the main text and supporting documents. The seismicity data recorded by the NASA InSight mission is available in the public domain from Clinton et al.^54^ and Knapmeyer et al.^18^. Emergent type Moonquakes data is available in the public domain from Dimech et al.^76^ and the deep and shallow Moonquakes from Galveston Geophysics Laboratory of the University of Texas (http://www-udc.ig.utexas.edu/external/yosio/PSE/catsrepts/).
