# Supplementary material for: Gravity-induced seismicity modulation on planetary bodies and their natural satellites
Source: Sci Rep. 2024 Jan 28;14:2311. doi: 10.1038/s41598-024-52809-7 (PMC10821896; doi:10.1038/s41598-024-52809-7)
Supplement: Supplementary file 1 — Supplementary Information. [file 41598_2024_52809_MOESM1_ESM.docx]

**Supporting Document**

**Gravity-induced seismicity modulation on planetary bodies and their natural satellites**

**Batakrushna Senapati^1^, Bhaskar Kundu^1*^, Birendra Jha^2^ and Shuanggen Jin^3,4^**

^1^Department of Earth and Atmospheric Sciences, NIT Rourkela, Rourkela, India, 769008

^2^Department of Chemical Engineering and Materials Science, University of Southern California, Los Angeles, CA 90007-1211, USA

^3^School of Surveying and Land Information Engineering, Henan Polytechnic University, Jiaozuo 454000, China

^4^Shanghai Astronomical Observatory, Chinese Academy of Sciences, Shanghai 200030, China

***Corresponding author:** Bhaskar Kundu, Department of Earth and Atmospheric Sciences, NIT Rourkela, Rourkela-769008, India, ([rilbhaskar@gmail.com](mailto:rilbhaskar@gmail.com))

**Material and Methods**

**Seismicity/tremors, Marsquakes and Moonquakes datasets**

We have considered several seismicity catalogues to analyse the modulation of seismicity by the external stress perturbation on Earth, Moon, and Mars. For Earth, we have analyzed the seismicity data surrounding the New Madrid seismic zone (NMSZ), which is retrieved from the Center for Earthquake Research and Information, <http://www.memphis.edu/ceri/seismic/catalog.php> for the period of 1974 to 2016. Non-volcanic tremors catalogue from South West Japan is archived from Slow Earthquake Database (<http://www-solid.eps.s.u-tokyo.ac.jp/~sloweq/>) for the period of 2003 to 2013. The tremors data from Cascadia for the period of 2005-2014 is archived from the Pacific Northwest Seismic Network operated and maintained by the University of Washington and the University of Oregon (<https://pnsn.org/tremor>). The Seismicity data surrounding the Delhi region from 2000-2020 is controlled by the National Center of Seismology, Delhi (NCS) and is available at <https://seismo.gov.in/>. Seismicity catalogue from the Nepal Himalaya taken from the National Seismic Centre, Nepal (<http://www.seismonepal.gov.np/>). The micro-seismicity data for the Juan De Fuca region are available at <http://ddrt.ldeo.columbia.edu/Axial>^1.^

For Moon, we have analyzed thermal Moonquakes data that are recorded by the Apollo 17 LSEP (Lunar Seismic Experiment Package) experiment module, which is available in the public domain^2^ and downloaded at <https://data.mendeley.com/datasets/g3yccthhwn/2>.The deep and shallow Moonquakes are recorded in Apollo Passive Seismic Experiment Long-Period Event Catalog, available at Galveston Geophysics Laboratory of the University of Texas and archived from <http://www-udc.ig.utexas.edu/external/yosio/PSE/catsrepts/>.

For Mars, the seismicity data recorded by the NASA InSight mission is available in the public domain^3-4^ and archived at <https://ars.els-cdn.com/content/image/1-s2.0-S0031920120302739-mmc2.pdf>.

**Magnitude completeness (Mc), b-value estimation and** **Declustering**

We have analyzed the Gutenberg-Richter relation [logN (M≥ Mc) = a − b × Mc] of the seismicity catalogues for the Nepal Himalayan, New Madrid seismic zone, Juan De Fuca, Delhi seismic zone, Nankai subduction zone, and Cascadia subduction zone by employing maximum likelihood approach^5^. In the case of the Nepal Himalayan and New Madrid seismic zone, the lower magnitude threshold (Mc) are 2 and 1.4, respectively. For Juan De Fuca, the Mc value is considered as 0.1. Similarly, the Mc for the Delhi seismicity region and Cascadia subduction zone consider as 2.5 and 0.1, respectively. Moreover, we have also generated an aftershock-depleted seismicity catalogue for both the New Madrid seismic zone, Nepal Himalayan, Juan De Fuca, and Delhi seismic zone using the approach from Reasenberg^6^, with P = 0.95 and relaxation time (τ), in the range of 1 and 10 days, assuming a horizontal location error of 5 km and vertical error in hypocentre location of 10 km.

**Periodicity analysis**

***Schuster Spectra analysis***

In order to estimate the periodicity of the marsquakes, we have computed the spectra of Schuster p-values by considering the methodology proposed by Ader and Avouac^7^. For the computation of probability, the timing of events ($t_{k}$) in a catalogue vary according to a sine-wave function of period T, which is associated with the phase ($\theta_{k}$) represented as $\theta_{k}=2\pi\frac{t_{k}}{T}$. Further, the probability ($p$) which arises due to the event-time distribution from a uniform seismicity rate can be expressed as^7^:

$p=e^{\frac{-D^{2}}{N}}$ (1)

where, $p$ is the Schuster probability value, $N$ is the number of events in the seismicity catalogue and $D$ is the time span between the start and end point of the seismicity catalogue^7^.

***Power Spectrum density analysis***

We have also analyzed the periodicity for Mars (B, C, and D type Marsquakes), Moon (Deep Moonquakes and Thermal Moonquakes) and Earth (Nepal Himalayan, New Madrid seismic zone, Juan De Fuca, Delhi seismic zone, Nankai subduction zone, and Cascadia subduction zone) using the Power Spectra density analysis (PDS) by adopted the methodology proposed by the Stoica & Moses^8^. Before PDS analysis, we have generated continuous seismicity time series by converting the entire period of the earthquake time series into the number of events per hour. The Power spectrum $S_{xx}\left( w \right)$ of a time series x(t) described the distribution of power into frequency components composing that signal and expressed as:

$P={\lim_{T\to\infty} \frac{1}{T}\int_{0}^{T} \left| x(t) \right|}^{2}dt$ (2)

$S_{xx}\left( w \right)=\lim_{T\to\infty} E\left[ \left| \hat{x} \left( w \right) \right|^{2} \right]$ (3)

where P= average power, x(t) = time series, *S_xx_* = power spectrum and E=energy of a signal

**Supporting Figures**


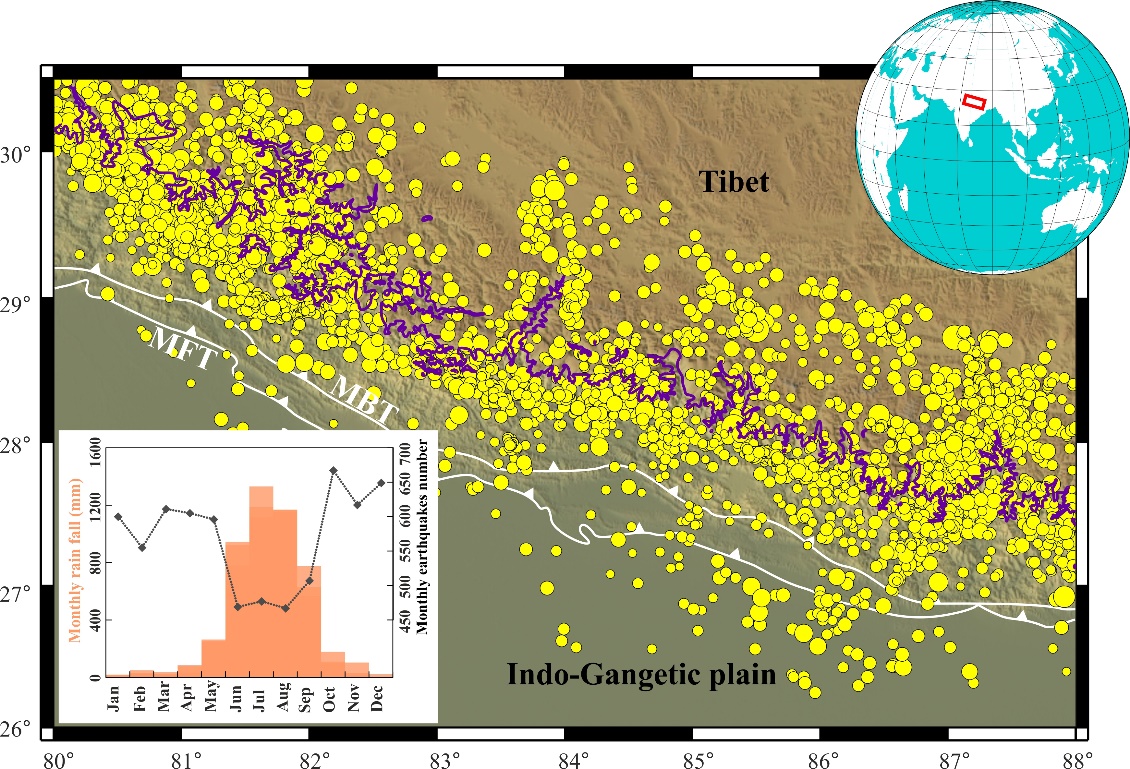


**Figure S1.** (a) Spatial distribution of mid-crustal seismicity (yellow circles). Inset shows the monthly stack of rainfall and mid-crustal seismicity. Note that the mid-crustal seismicity decreases with increasing of rainfall, which indicates seasonal deformation.

**Note:** The rainfall of the Nepal Himalayan region is archived at the Tropical Rainfall Measuring Mission (TRMM, <https://gpm.nasa.gov/missions/trmm>) and the microseismicity catalogue associated with the Nepal Himalaya is archived from the National Seismic Centre, Nepal (<http://www.seismonepal.gov.np/>).

The seismicity of the Nepal Himalayan region reduces during the rainy season and increases during the summer season (Fig. S1). Hence, the microseismicity associated with the Nepal Himalayan region is mainly modulated by seasonal hydrological loading.


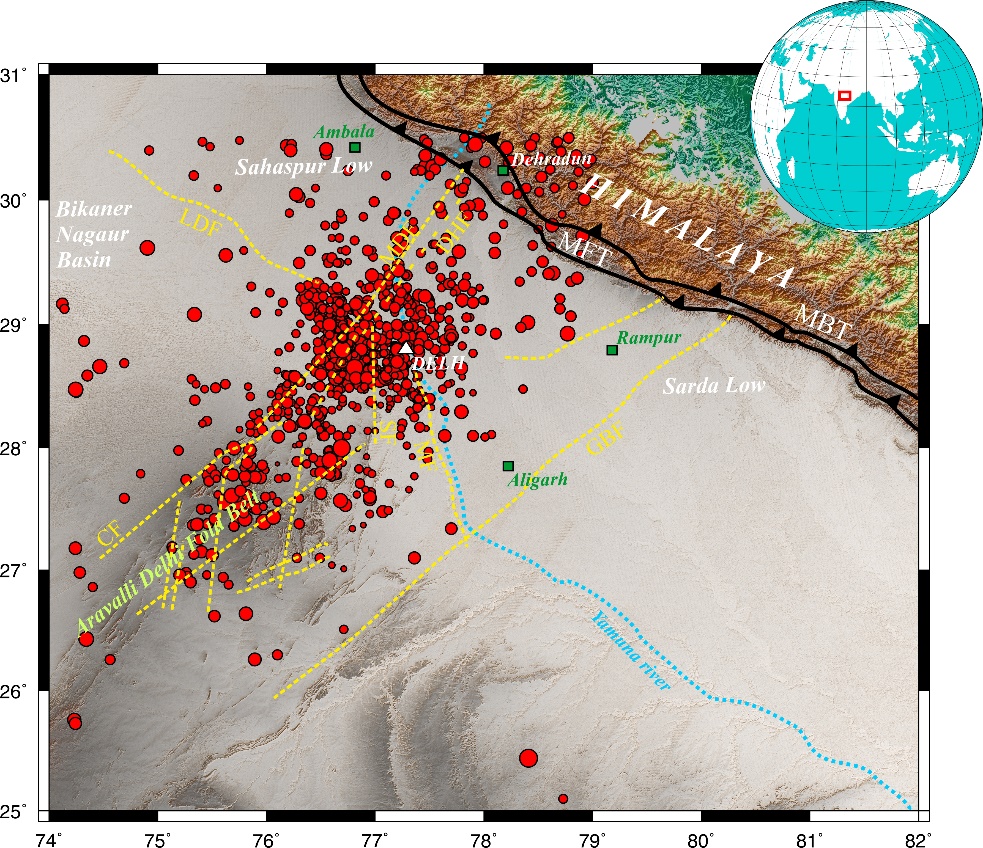


**Figure S2.** (a) Epicentral distribution of seismicity associated with Delhi and its surrounding regions for the periods of 2000-2020. Different basement faults are marked by dashed lines. MDF-Mahendragarh Dehradun Fault, CF-Chahapoli Fault, SF-Sohna Fault, MF-Mathura Fault, GBF-Great Boundary Fault, DHF-Delhi Haridwar Fault, MBT-Main Boundary Thrust, MFT-Main Frontal Thrust (Taken from Tiwari et al.^9^).

**Note:** The seismicity associated with Delhi and its surrounding regions for the periods of 2000-2020 is controlled by the National Center of Seismology, Delhi (NCS) and is available at <https://seismo.gov.in/>. The topography data is downloaded from the <https://www.gmrt.org/GMRTMapTool/>


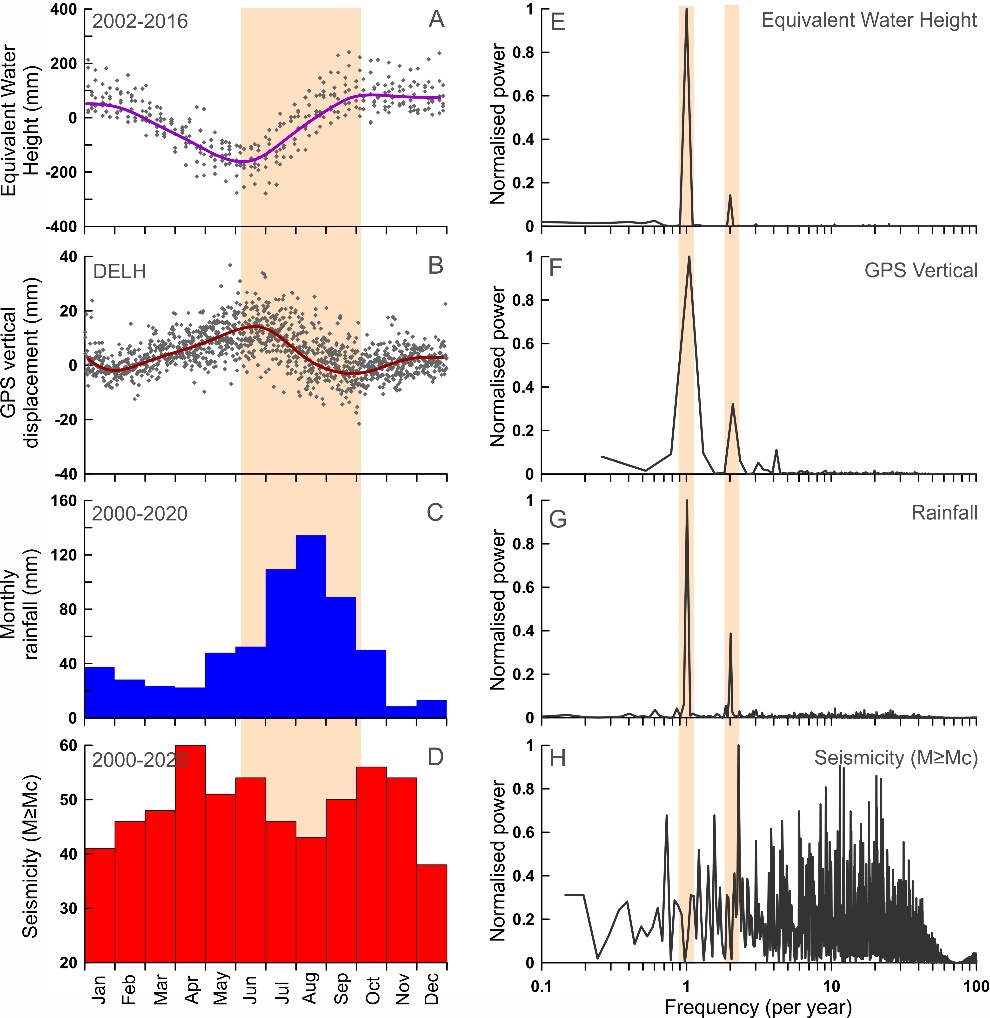


**Figure S3.** (*Left panel*) Monthly stacked time series of the equivalent water height, GPS vertical displacement (station DELH), rainfall derived from TRMM, and monthly seismicity (M≥Mc). Orange strip represents the hydrological loading period. (*Right panel*) Power spectrum analysis of equivalent water height, GPS displacement, rainfall and seismicity catalogue from Aravalli Delhi fold belt region. Note that equivalent water height, GPS displacement and rainfall show strong annual and weak semi-annual periodicity, whereas seismicity associated with the Aravalli Delhi fold belt region exhibits strong semi-annual periodicity (Taken from Tiwari et al.^9^).

**Note:** The GPS time series from the Delhi region is archived from <http://geodesy.unr.edu/NGLStationPages/gpsnetmap/GPSNetMap.html>. GRACE data is archived from [http://grgs.obs-mip.fr/grace/variable-models-grace-lageos/grace-solutions- release-03](http://grgs.obs-mip.fr/grace/variable-models-grace-lageos/grace-solutions-%20release-03) for the period of 2002-2016. The rainfall is derived from TRMM (<https://disc.gsfc.nasa.gov/datasets/TRMM_3B42_7/summary>).

The time series of GRACE-derived equivalent water height (EWH), GPS-derived vertical displacement, regional rainfall, and seismicity (of M≥Mc), clearly shows that the earthquake occurrences have a good correlation with the timing of the seasonal hydrological loading cycle in the Delhi surrounding region (Fig. S3A-D).

We have also computed periodicity using the Power spectrum analysis of various physical parameters (Fig. S3E-H). From this analysis, it has appeared that equivalent water height (EWH), GPS-derived vertical displacement, and regional rainfall exhibit strong annual periodicity along with relatively weak semi-annual periodicity (Fig. S3E-H). Interestingly, the seismicity of the Delhi region clearly exhibits strong semi-annual periodicity. Moreover, during the seasonal loading period (June-September, i.e., during monsoon), the seismicity is lowest, whereas the seismicity level is relatively high during the unloading period.

Therefore, we propose that the precipitated water load during the monsoon period and seasonal recharge of the regional aquifer stabilize the causative faults in the basements, but the same faults are destabilized during the seasonal unloading period. This seasonal modulation of seismicity prompted us to explore whether the increased extraction of the groundwater (i.e., long-term decadal unloading) also influences the seismicity in the region.


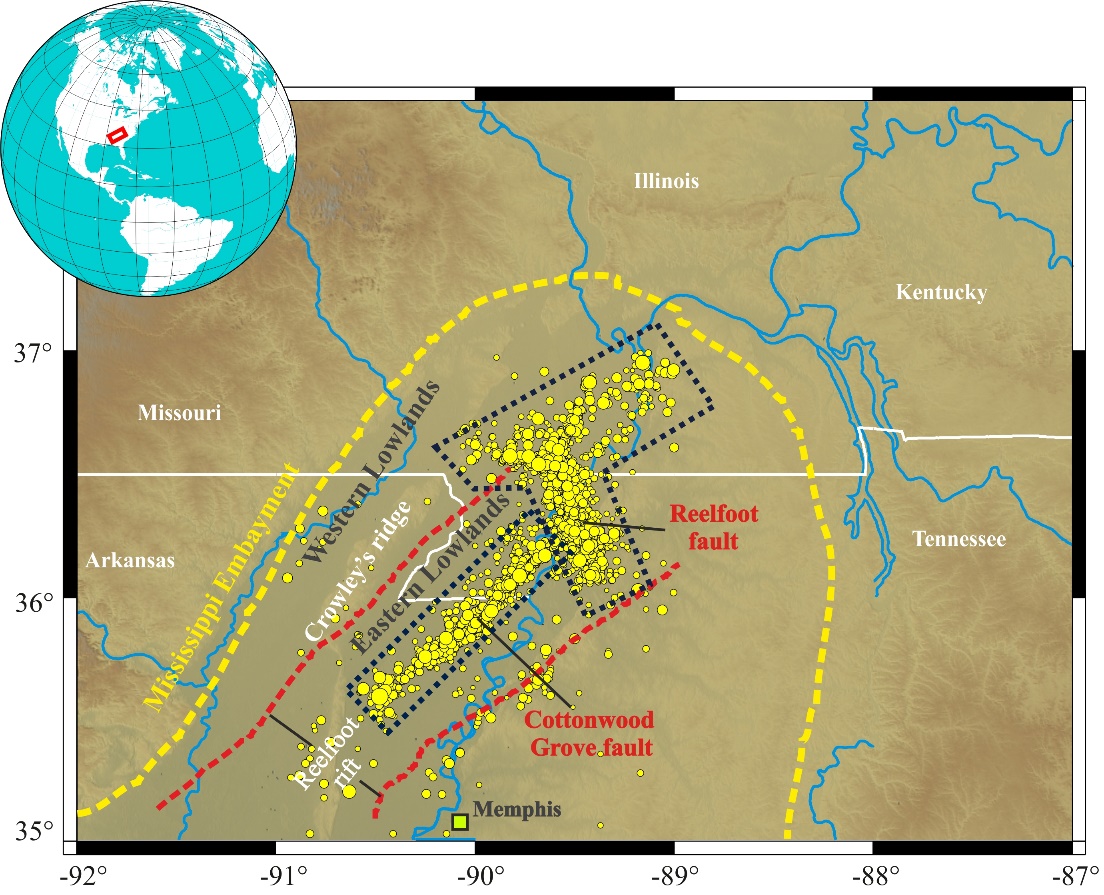


**Figure S4.** Epicentral distribution of declustered seismicity of Mc=1.4 (yellow circles), for the period of 1974-2016. The two red dotted lines mark the approximate extent of the Palaeozoic Reelfoot rift. Reelfoot and Cottonwood Grove fault zones are outlined by dashed polygons. The yellow dashed line shows the region of the Mississippi embayment.

**Note:** The Seismicity data surrounding the NMSZ is retrieved from the Center for Earthquake Research and Information, <http://www.memphis.edu/ceri/seismic/catalog.php> for the period of 1974 to 2016. The Seismicity of NMSZ is mainly associated with two principal structures, the Reelfoot thrust fault (considered a step-over arm) and the right-lateral Cottonwood Grove fault (Fig. S4). The Reelfoot fault is the most seismically active structure that produced several microseismic swarms and repeating earthquakes^10^. Seismic instrumentation and network geometry had evolved since 1974, when instrumental monitoring was initiated in the region. Since 2000, the overall network coverage has remained relatively stable^11^.


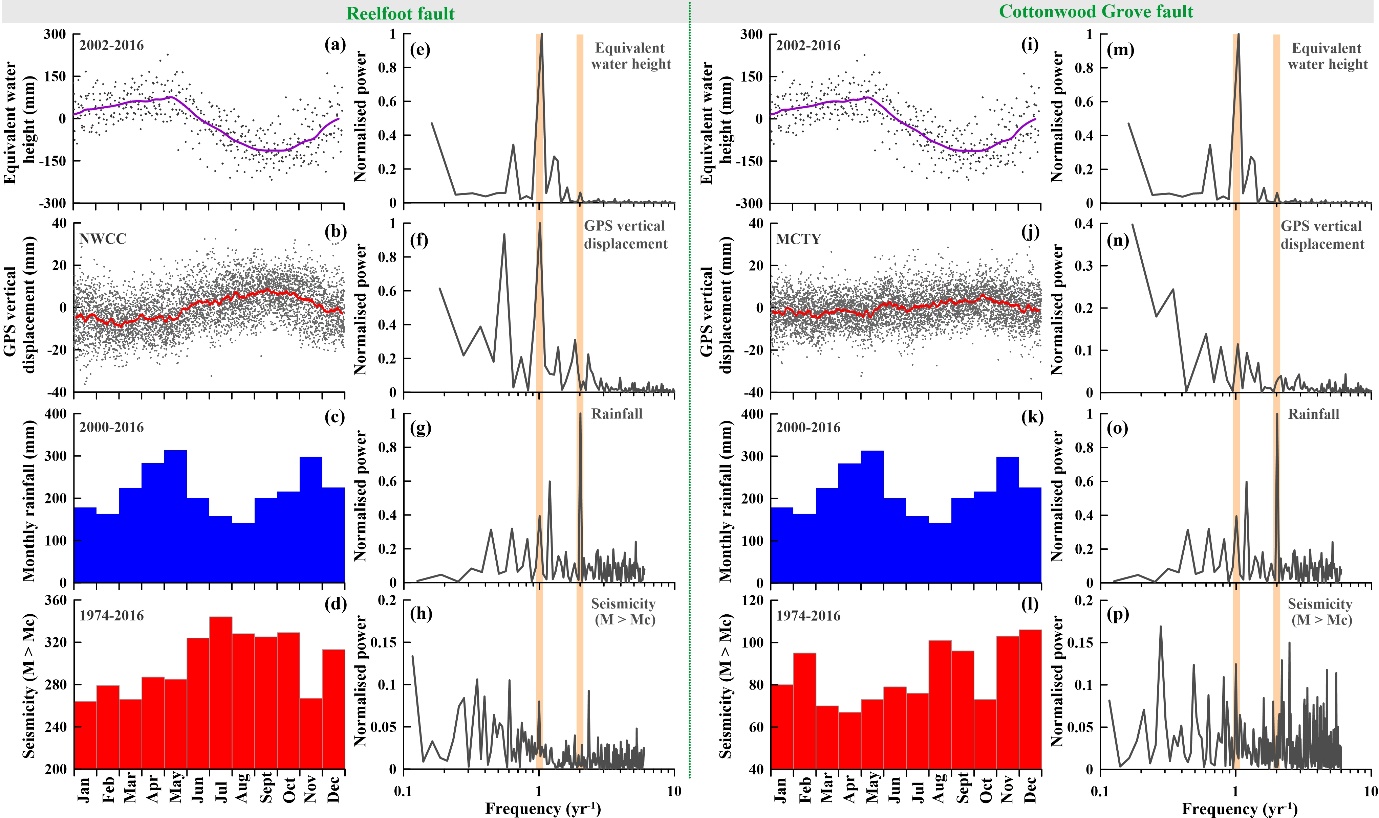


**Figure S5.** (*Left panel*) Monthly stacked time series of the equivalent water height, GPS vertical displacement (station NWCC), rainfall, and monthly decluster seismicity (Mc=1.4) for the Reelfoot fault. Power spectrum analysis of equivalent water height, GPS displacement, rainfall and seismicity catalogue from the Reelfoot fault region. (*Right panel*) Same as the Left panel but for Cottonwood Grove fault zones. Note that both the Reelfoot fault and Cottonwood Grove fault zones exhibit annual periodicity.

**Note:** The time series of GRACE-derived equivalent water height (EWH), GPS-derived vertical displacement, regional rainfall, and seismicity (of M≥Mc), clearly shows that the earthquake occurrences have a good correlation with the rainfall. From these time series, it has been observed that the maximum occurrences of the earthquake in the Reelfoot fault during the dry seasons (Fig. S5a-d), whereas no such correlation is observed in the case of the Cottonwood Grove fault.

We have also analyzed the periodicity of the EWH, GPS-derived vertical displacement, regional rainfall, and seismicity (of M≥Mc) using Power spectrum analysis. It has been observed that the seismicity associated with NMSZ shows prominent annual frequency, which coincides with annual periodicities of GPS, EWH, and rainfall time series Fig. S5e-h). This suggested that the annual periodicity in the NMSZ seismicity is mainly modulated by seasonal hydrological loading.


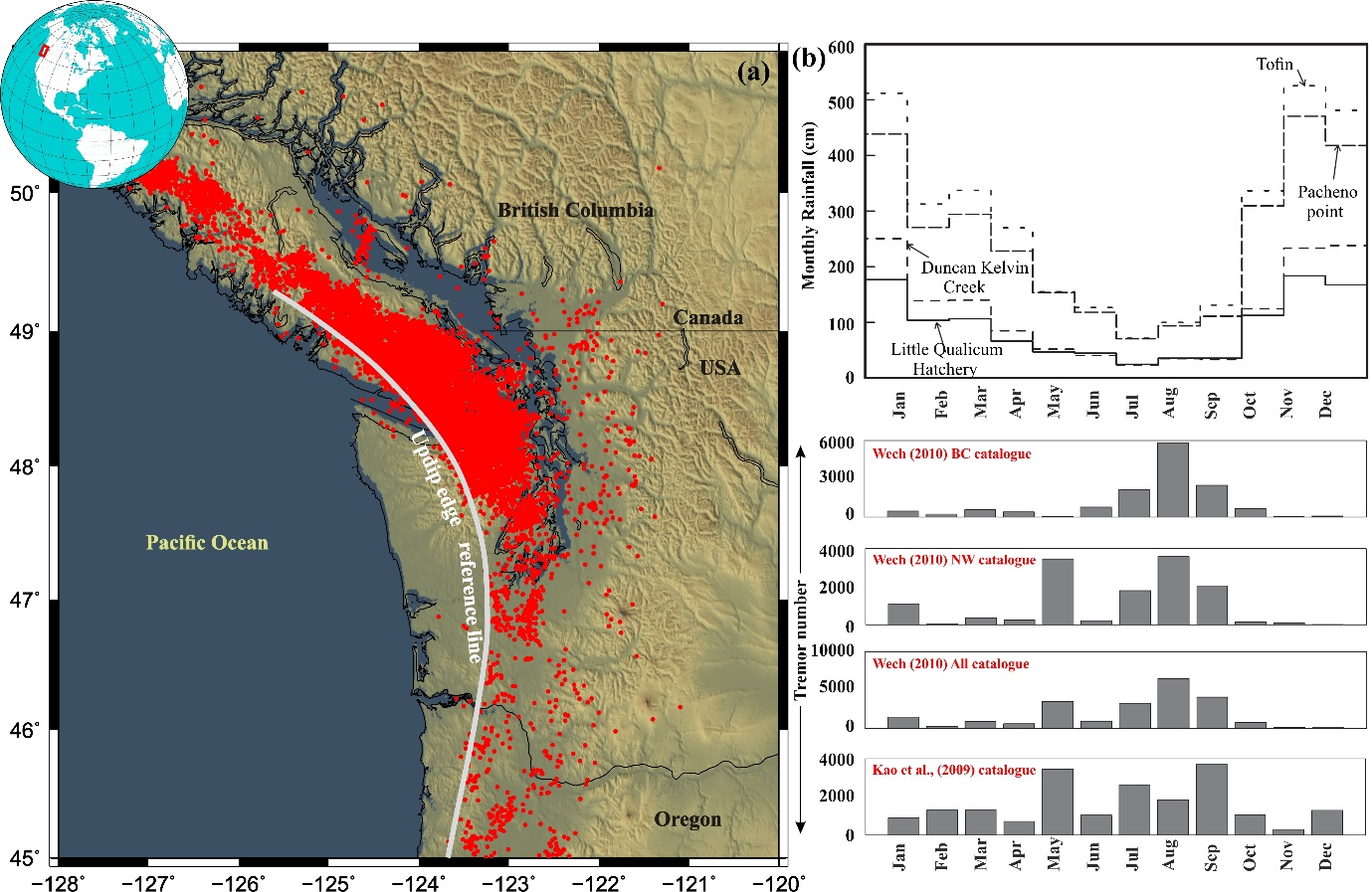


**Figure S6.** (*Left panel*) Epicentral distribution of tremors in the Cascadia subduction zone for the period of 2005 to 2014 (red dots). The up-dip edge of the tremor region is marked by a white line. (*Right panel*) Monthly rainfall and histogram of the different catalogue of the tremors from the Cascadia subduction zone. Note that the occurrence of tremors and rains shows a good correlation (i.e., higher seismicity occurred during the dry season) (Modified from Pollitz et al.^12^).

**Note:** The tremors data from Cascadia for the period of 2005-2014 is archived from the Pacific Northwest Seismic Network at <https://pnsn.org/tremoro>, operated and maintained by the University of Washington and the University of Oregon.

From the monthly histogram of the Rainfall and tremor numbers of the Cascadia subduction zone, it has observed the maximum number of tremors occurred during the dry season (Fig. S6b). This suggested that the tremor associated with the Cascadia subduction zone is mainly modulated by the hydrological load.

**
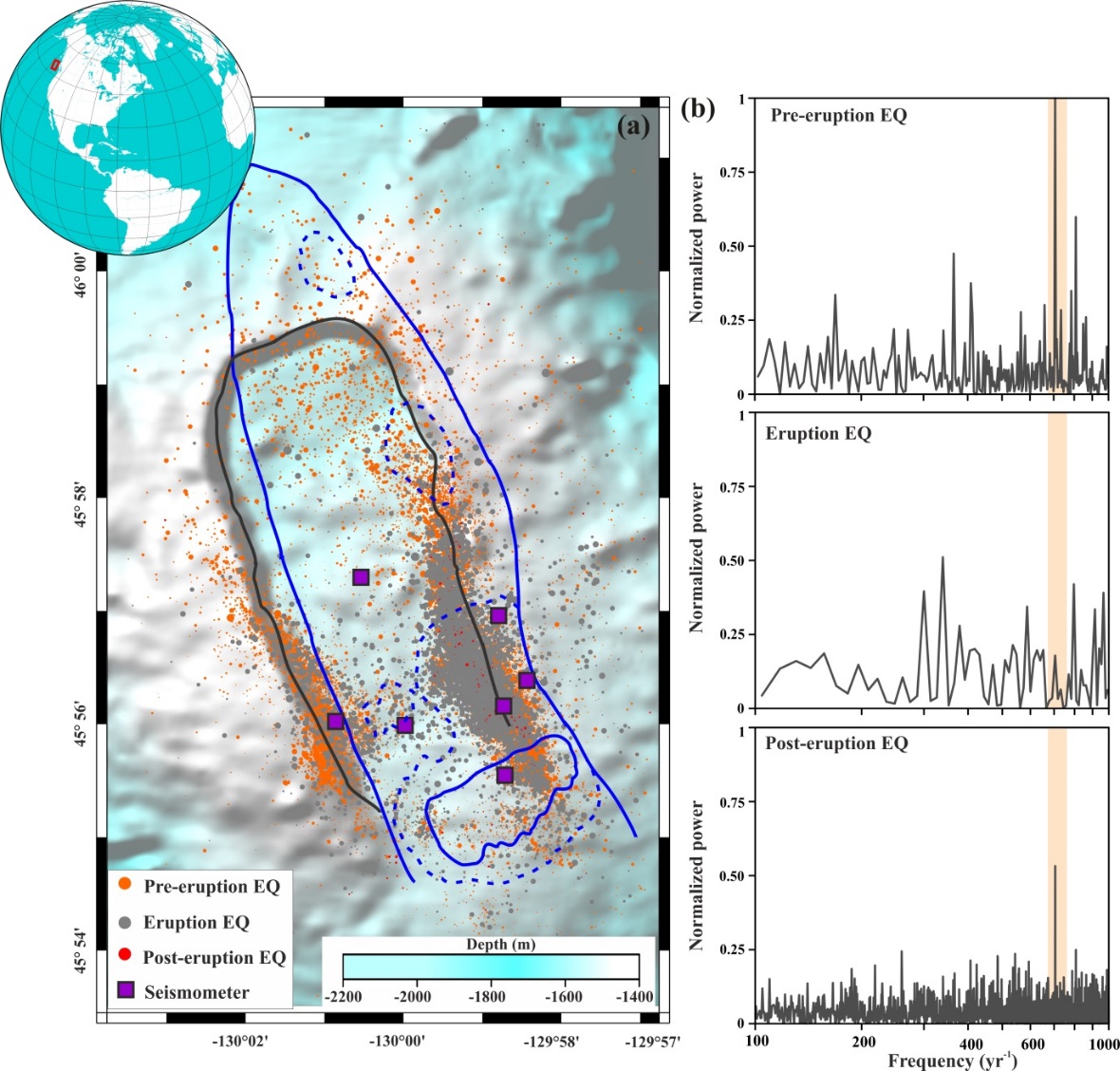
**

**Figure S7.** (a) Bathymetry and spatial distribution of micro-seismicity, seismic network and collocated bottom pressure recorders (purple squares) surrounding the Axial Seamount on the Juan de Fuca ridge. The seismicity is divided into pre-eruption, eruption and post-eruption. (b) Power spectrum analysis of Declustered micro-seismicity (with Mc 0.1) during three phases of the eruption. Seismicity during the pre-eruption phase shows a strong semi-diurnal phase. While seismicity during the eruption phase has an absence of a semi-diurnal phase, and seismicity during the Post-eruption phase shows a weak semi-diurnal periodicity (Modified from Sahoo et al.^13^).

**Note:** The micro-seismicity data for the Juan De Fuca region are available at <http://ddrt.ldeo.columbia.edu/Axial>^1^. The bathymetry data is archived from the General Bathymetric Chart of the oceans (<https://www.gmrt.org>). The tide gauge station (yellow triangle) at the coast of the Cascadia Subduction zone is archived at <https://maps.ngdc.noaa.gov/viewers/hazards/>).

We have analyzed the Gutenberg-Richter relation [logN (M≥ Mc) = a − b × Mc] of the micro-seismicity associated with the Juan De Fuca region and found that the lower magnitude threshold (Mc) is about 0.1. Moreover, we have also declusted the seismicity is using the approach from Reasenberg^6^, with P = 0.95 and relaxation time (τ), in the range of 1 and 10 days, assuming a horizontal location error of 5 km and vertical error in hypocentre location of 10 km. We have analyzed the periodicity of the three phases of seismicity (i.e., pre-eruption, eruption and post-eruption) using power spectra analysis (Fig. S7b). From this analysis, it has been observed that the seismicity associated with the pre-eruption phase of Magma exhibits a strong semi-diurnal tidal period (M2), whereas the seismicity during the post-eruption phase shows a weak semi-diurnal tidal periodicity (Fig.S7b). The seismicity associated with the eruption phase of magma does not exhibit any tidal periodicity.

Therefore, we suggested that During the pre-eruption stage, fault systems are critically stressed and hence are more sensitive to stress perturbation by external periodic tidal loading. Therefore, prominent semi-diurnal tidal periodicities is observed in the micro-seismicity associated with the pre-eruption stage. However, during the eruption stage, volcano-tectonic processes dominate, which results in masking tidal modulations in the micro-seismicity.


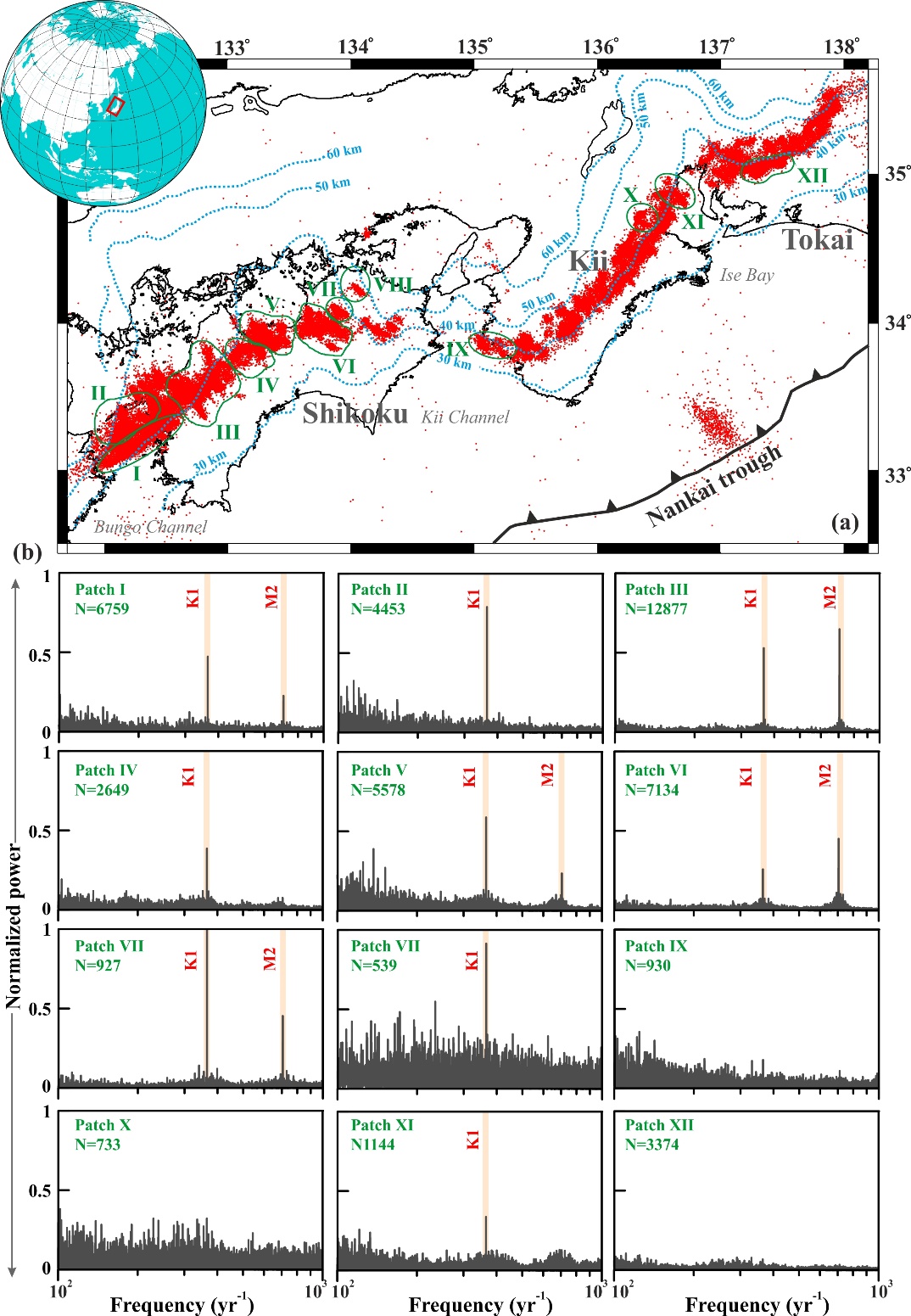


**Figure S8.** (a) Epicentral distribution of non-volcanic tremors (NVT) in Southwest Japan spanning from 2003 to 2013. Red dots are hourly centroid locations of tremors. Isodepth contours of the oceanic Moho discontinuity, derived from receiver function analysis^14^, are plotted as blue dashed lines. Green polygons (segment I-XI) are different tremor patches that are considered for further analysis. Inset shows the map of Japan with the locations of active volcanoes (red triangles). The blue box denotes the study region considered in this work, i.e., the South West coast of Japan, which has been enlarged. (b) Power spectrum analysis for NVT patch (I, III, IV, and VI) is marked in Fig. a (Power spectrum for all NVT patches (i.e., I-XII) shown in Fig. S7). Different tidal phases (M2 and K1) are marked by orange strips. M2-principal lunar (12.42 solar hrs period) and K1-luni-solar diurnal (23.93 solar hrs period) Note, the tidal periodicity is different for different patches, indicating heterogeneous behavior of individual tremor patches (Taken from Senapati et al.^15^).

**Note:** Non-volcanic tremors catalogue from South West Japan is archived from the Slow Earthquake Database (<http://www-solid.eps.s.u-tokyo.ac.jp/~sloweq/>) for the period of 2003 to 2013.

Here, we have examined the possible influence of tidal loading on the occurrence of well-monitored non-volcanic tremor (NVT) in the Nankai subduction zone, South West Japan (Fig. S8a). The NVT patches occur in a narrow zone belt from the center of Honshu to western Shikoku at ~30 km depth on the subducting interface of the Philippine Sea plate below South West Japan (Fig. S8a).

To explore the tidal periodicity for the NVT, we have presented power spectra analysis for various tremor patches (marked as Patch I-XII, in Fig. S8a and b). It has clearly been noticed that the NVT patches exhibit significant semi-diurnal (M2) and diurnal periodicity (K1) (Fig. S8b). The variation of tidal periodicity in these NVT patches reflects the heterogeneous nature of inter-plate frictional properties (Fig. S8b).

In addition to this, Kodaira et al.^16^ have identified anomalous pore fluid pressure along the non-volcanic tremor patches. Hence, we suggest that the anomalous pore fluid pressure along the NVT patches makes the interplate segment more sensitive to periodic stress perturbations by tidal loading and modulates the tremors associated with the Nankai subduction zone in South West Japan.


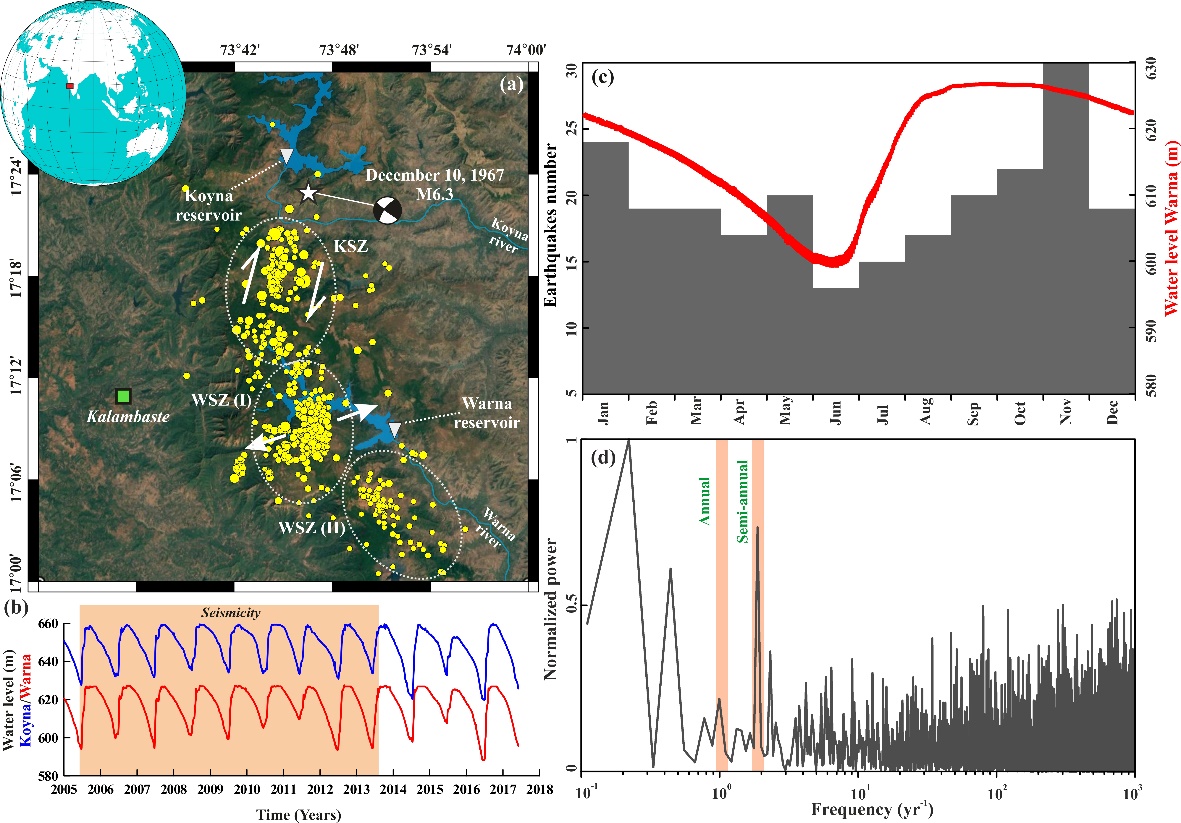


**Figure S9.** (a) Spatial distribution of the declustered seismicity catalogue of Mc>2.0 (yellow circles) from the Koyna-Warna region for the time period of 2005-2013, White star indicates the location of the M6.3 earthquakes along with its focal mechanism solution. The entire zone is divided into three segments based upon the spatial distribution of seismicity clusters, i.e., Koyna Seismic Zone (KSZ), Warna Seismic Zone (I), and Warna Seismic Zone (II). Inverted white triangles indicate the locations of the Koyna and Warna dam sites. (b) The lower panel represents the water level variation of the Koyna (red line) and Warna (blue line) reservoirs. The light-shaded region represents the duration of the seismicity catalouge. (c) Monthly stacked time series of water level variation in Warna reservoir (red curve) and monthly histogram of decluster seismicity associated with Warna Seismic Zone (I). (d) Power spectrum analysis of decluster seismicity catalogue (Mc>2.0) from the WSZ (I)Note that WSZ (I) shows strong annual and semi-annual periodicity (Modified from Senapati et al.^15^).

**Note:** The Seismicity catalogue from the Koyna-Warna region region is taken from the local seismological station network operated and maintained by CSIR, NGRI, Hyderabad. Water level data from the Koyna and Warna reservoir is obtained from the Division of Koyna and Warna Dam authorities, Maharashtra, during the period of 2005-2017.

The Koyna-Warna seismic zone represents three distinct clusters of seismicity, namely, Koyna Seismic Zone (KSZ), Warna Seismic Zone I (WSZ I), and Warna Seismic Zone II (WSZ II) (Fig. S9a). Seismic instrumentation and network geometry have changed and improved since the occurrence of the M6.3, 1967 Koyna earthquake. However, since 2005, the overall network geometry has remained stable, allowing us to exploit an earthquake catalogue from 2005-2013 (with a lower magnitude threshold Mc=2) together with reservoirs' water level data^17^.

Here, we have correlated the seismicity rate variation in WSZ I with reservoir water level variation in Koyna- Warna region (Fig. S9c). From this analysis, it has been observed that the seismicity rate in the WSZ I increases with the increasing water level in the reservoirs (Fig. S9c).

We have also performed power spectra analysis for declustered seismicity catalogues for the three distinct seismicity clusters of the Koyna-Warna region by converting the entire period of the earthquake time series into the number of events per hour. From the periodicity analysis, we observe that the seismicity associated with WSZ I exhibits statistically significant annual and semi-annual periodicity (Fig. S9d). However, we have not observed any tidal periodicity in this region, although this region is situated very near to the Coast, where we should expect high tidal stress (Fig. S13d). Therefore, we suggested that the annual variation of the reservoir water level appears to modulate the seismicity in the fluid-filled region of WSZ I.


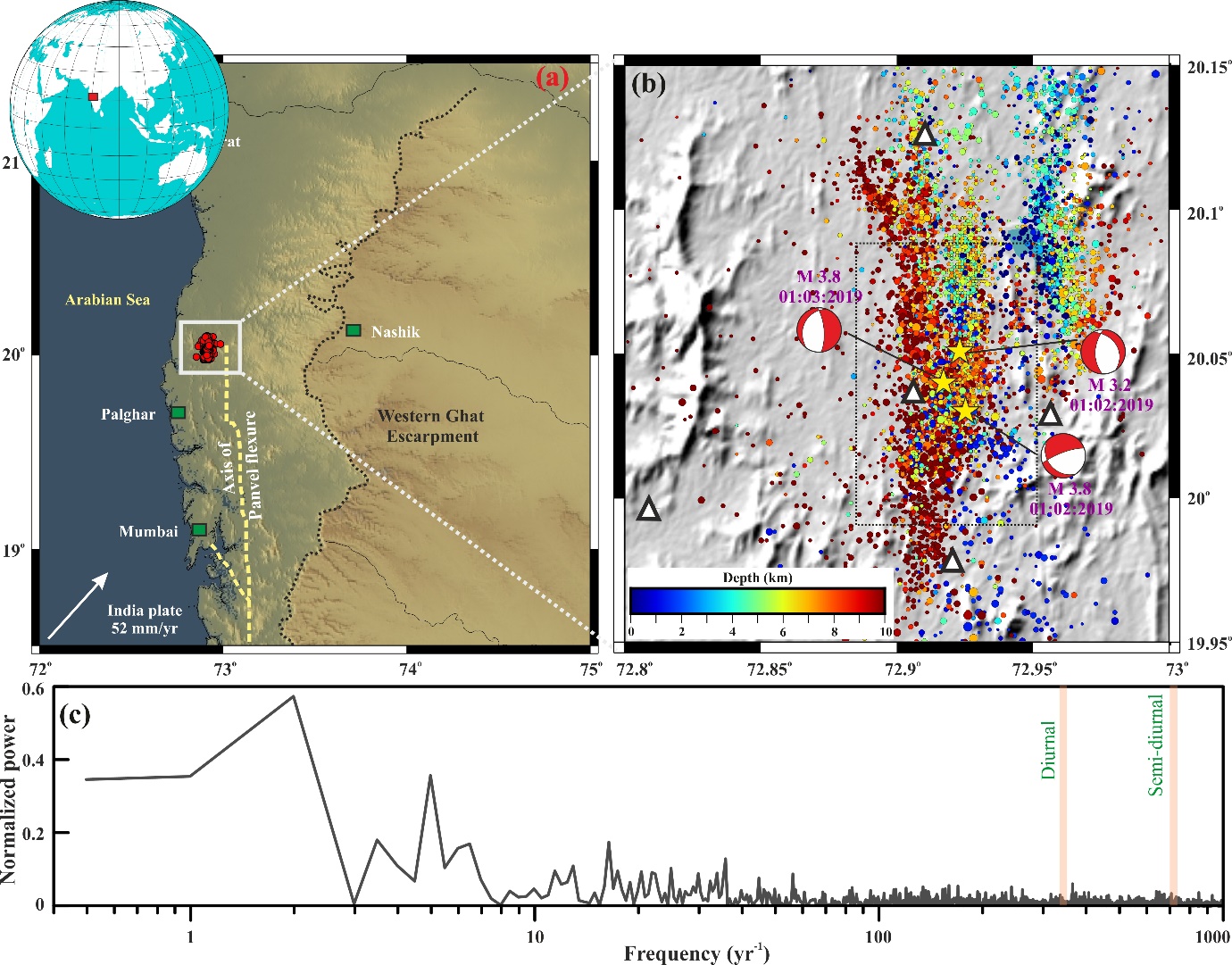


**Figure S10.** (a) Location of Palghar swarm (white square) and epicentral distribution of raw seismicity (Mc=1.2) seismicity for the periods of 2019-2020. The yellow dashed represents the axis of Panvel flexure and the direction of India plate motion is shown by white arrow. (b) Zoomed version of Palghar swarm with the depth-wise distribution of seismicity and focal mechanisms of representative earthquakes (yellow stars). Seismic network stations and magnetotelluric sites are marked by white triangles and black triangles, respectively. (c) Power spectra analysis for the raw seismicity (Mc=1.2) of the Palghar swarm. Note that the seismicity associated with the Palghar swarm does not show any tidal periodicity.

**Note:** The Seismicity catalogue from the Palghar swarm region is available in the published literature^18^ and can be downloaded from <https://link.springer.com/article/10.1007/s10950-022-10087-8#Sec6>. Here, we have performed power spectra analysis for declustered seismicity catalogues associated with the Palghar swarm. It has been observed that the seismicity associated with the Palghar swarm does not exhibit any tidal periodicity (Fig. S10). However, the seismicity of this region shows a good correlation with the rainfall^18^.


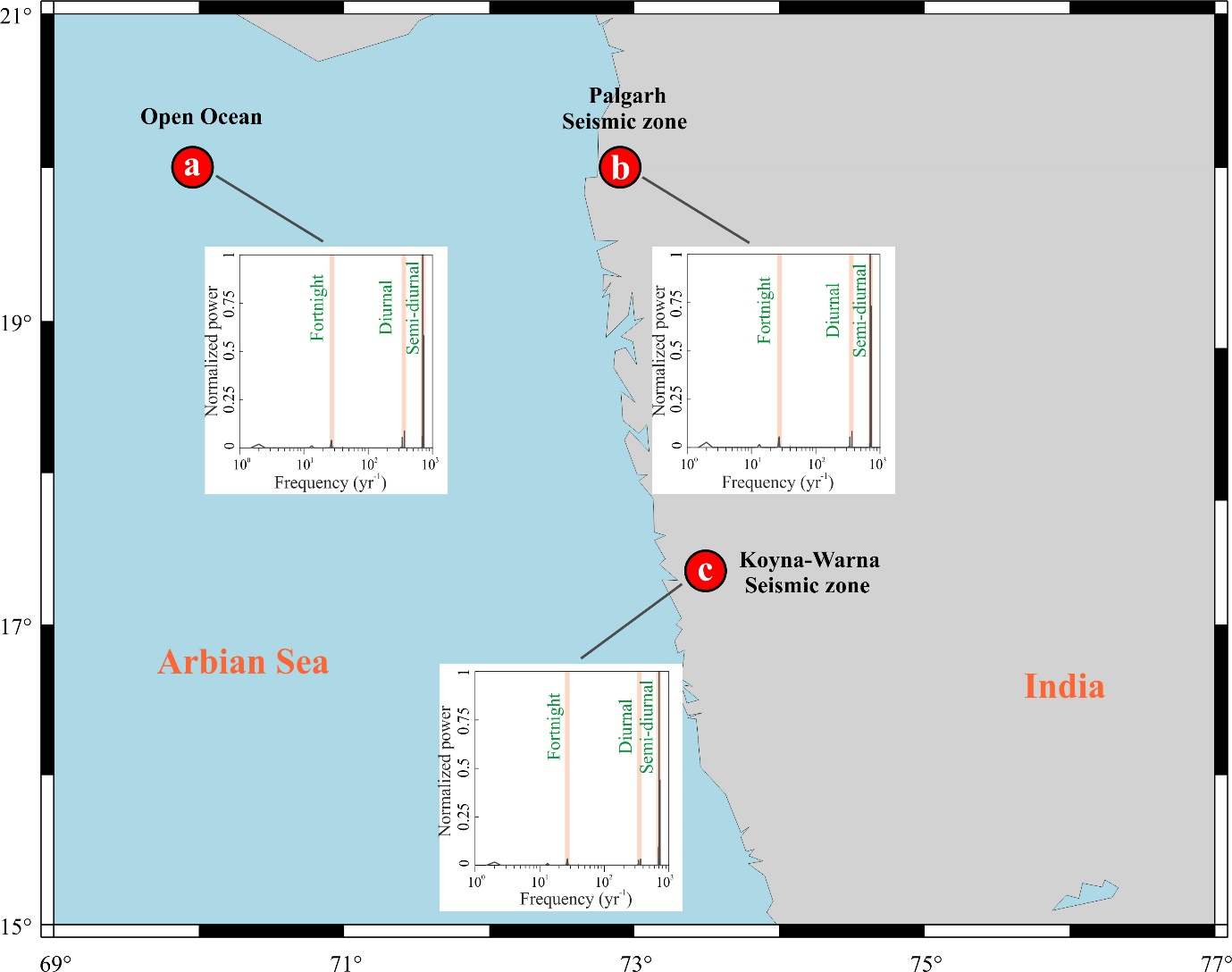


**Figure S11.** (a) Location of the Palghar Seismic zone, Koyna-Warna Seismic zone, Open Ocean point, at which stress is calculated. The inset figures represent the power spectra analysis of the tidal stress at respective points (shown in red circles). Note that tidal stress exhibits a strong semi-diurnal and a weak diurnal periodicity at all these locations.

**Note:** Here, we have calculated tidal stress at the Palghar Seismic zone, Koyna-Warna Seismic zone and open Ocean point (shown in Fig.S11) using the SPOTL program. We have also performed power spectra analysis to estimate the periodicity of the tidal stress at the Palghar Seismic zone, Koyna-Warna Seismic zone, and open Ocean point, respectively. From this analysis, it is observed that the tidal stress exhibits a strong semi-diurnal phase and a weak diurnal and fortnight phase (Fig.S11).


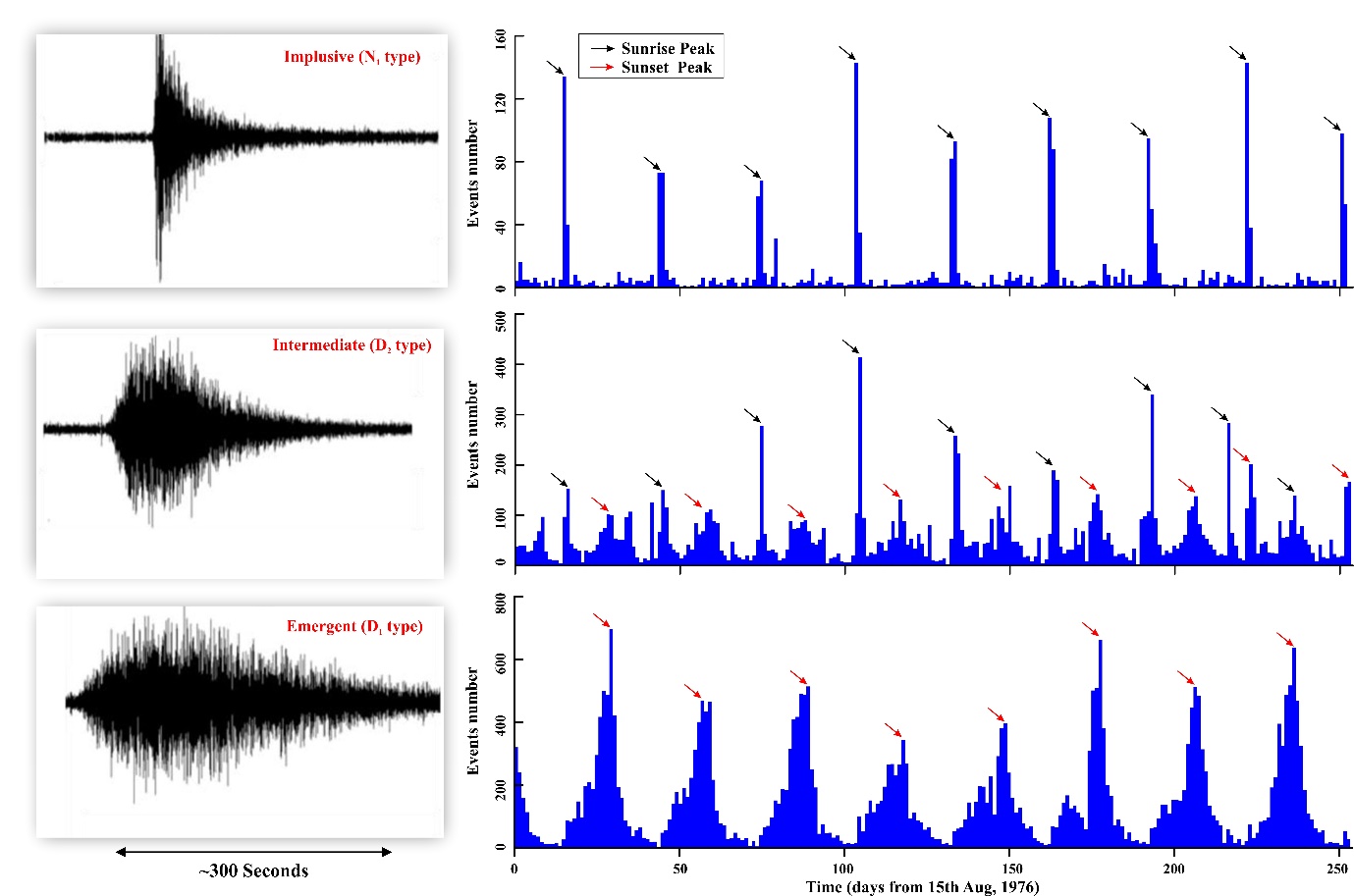


**Figure S12.** Histogram of all the seismic events Impulsive, Intermediate and Emergent type recorded over a period of 8.3 months on Apollo 17 ALSPE experiment. *Left panel*: Seismic event templates cut from Apollo 17 seismic data using by the HMM pattern recognition^2^.

**Note:** The thermal Moonquakes data that are recorded by the Apollo 17 LSEP (Lunar Seismic Experiment Package) experiment module are available in published literature^2^ and downloaded at <https://data.mendeley.com/datasets/g3yccthhwn/2>.

The Apollo 17 geophones, which were a part of the Lunar Seismic Experiment Package (LSEP), recorded 49,635 moonquakes. These moonquakes were classified based on their waveforms into three types, out of these, 2249 (4.5%) are impulsive type events, and 13,355 (26.9%) are intermediate type events, and 34,031 (68.5%) are emergent types^2^. The daily histogram of these events shows that impulsive type events are occurred during both Sunrise and Sunset times, whereas, the emergent types occur mainly during the sunset time (Fig. S12).

The impulsive events possibly originate from the heating of the Apollo 17 lunar lander, while emergent events are from the gradual heating of regional soil and rocks, which experience strain due to thermal contraction and expansion (Fig. S12).


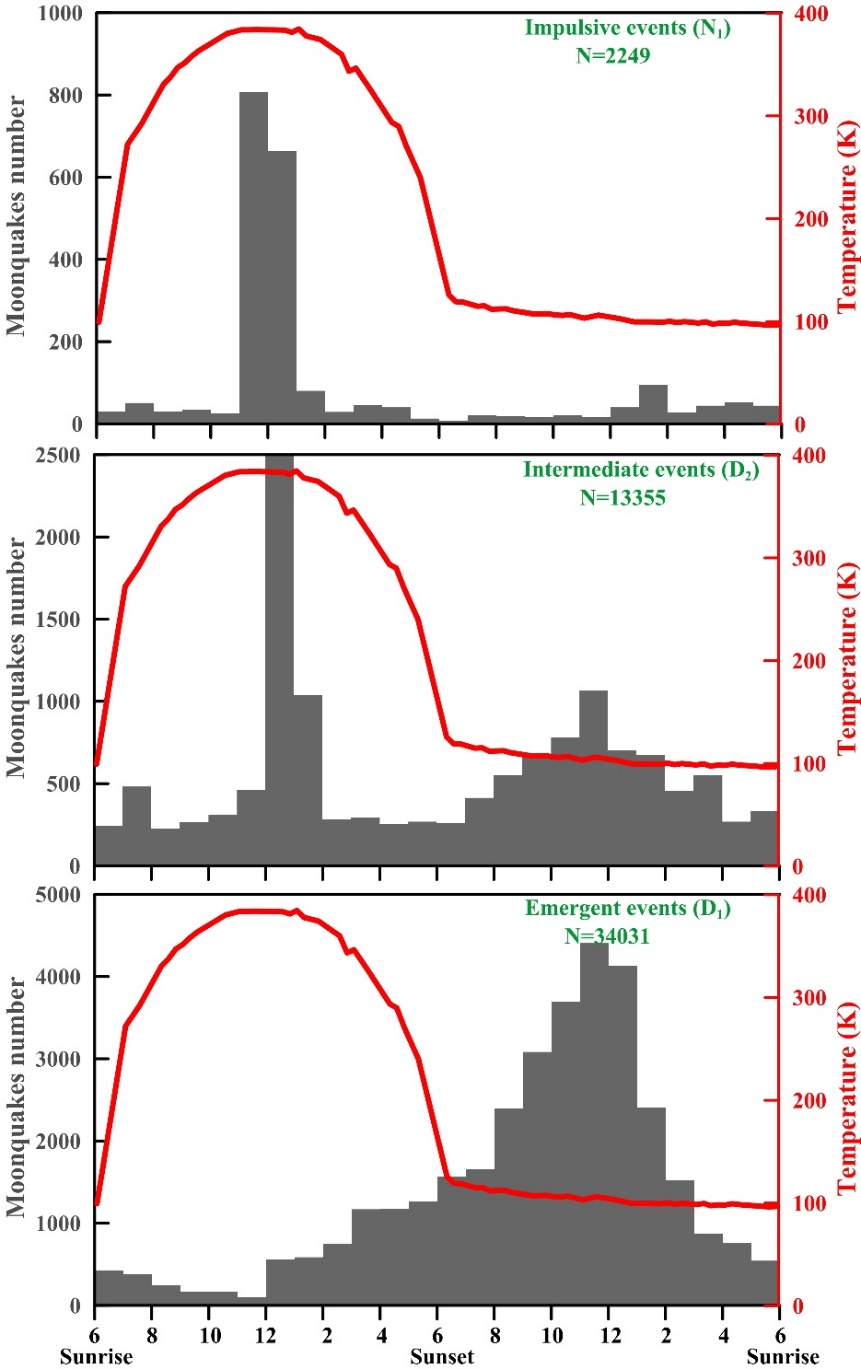


**Figure S13.** Lunar Surface Temperature (Red) and histogram of moonquakes for Impulsive (Top), Intermediate (Middle) & Emergent (Bottom) events. Note that the maximum occurrence of Impulsive and Intermediate Moonquakes during the noon time, whereas Emergent events occurred during sunset time.

**Note:** The temperature data of the Moon's is controlled by the Geosciences Node of NASA's Planetary Data System (PDS), which can be archived from (<https://pds-geosciences.wustl.edu/missions/apollo/index.htm>).

The Thermal moonquakes show a good correlation with the temperature of the Moon's surface (Fig.S10). The impulsive events possibly originate from the heating of the Apollo 17 lunar lander, while emergent events are from the gradual heating of regional soil and rocks, which experience strain due to thermal contraction and expansion (Fig. S13).

**
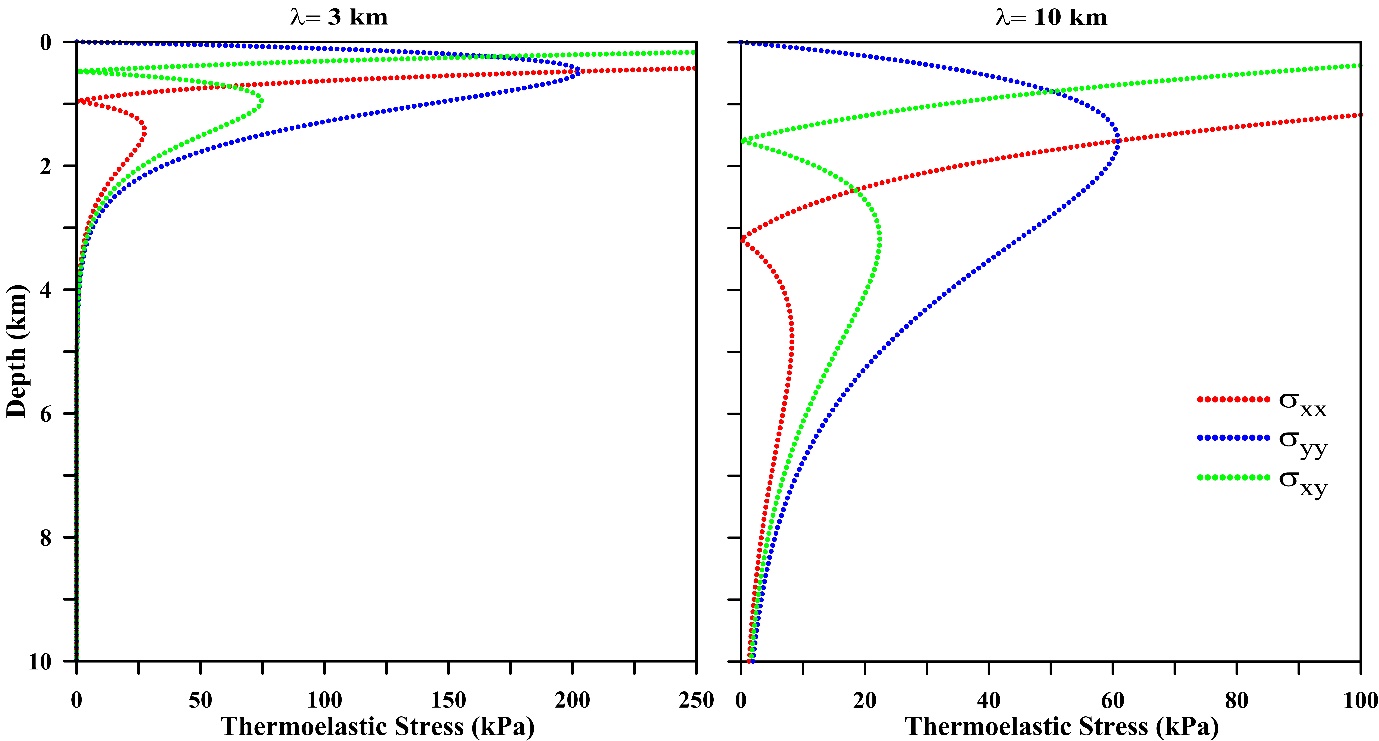
**

**Figure S14.** Thermoelastic stress is induced by a temporal and spatial variation in surface temperature in a half-space. Amplitudes of thermoelastic stress for the model induced by a temperature variation at the surface, with a period of 24 hours, an amplitude of 290°C, a spatial wavelength of 3 km and 10 km, respectively. Other parameters used in this model are shown in the inset table. Note that the Thermoelastic stress is about ~200 kPa at a depth range 0-5 km, which can be capable of modulating the moonquakes.

**Note:** The daily temperature variation of the Moon's surface is about 286 °C, hence using the temperature difference in the Moon's surface, we calculated the thermoelastic stress in a half-space. The other parameters used to calculate the thermoelastic stress are shown in the table (Fig. S14). From this analysis, it has been observed that thermoelastic stress varies at a range ~200 kPa at a depth 0-5km (Fig. S14), which is probably sufficient to modulate the seismicity on the Moon's surface.


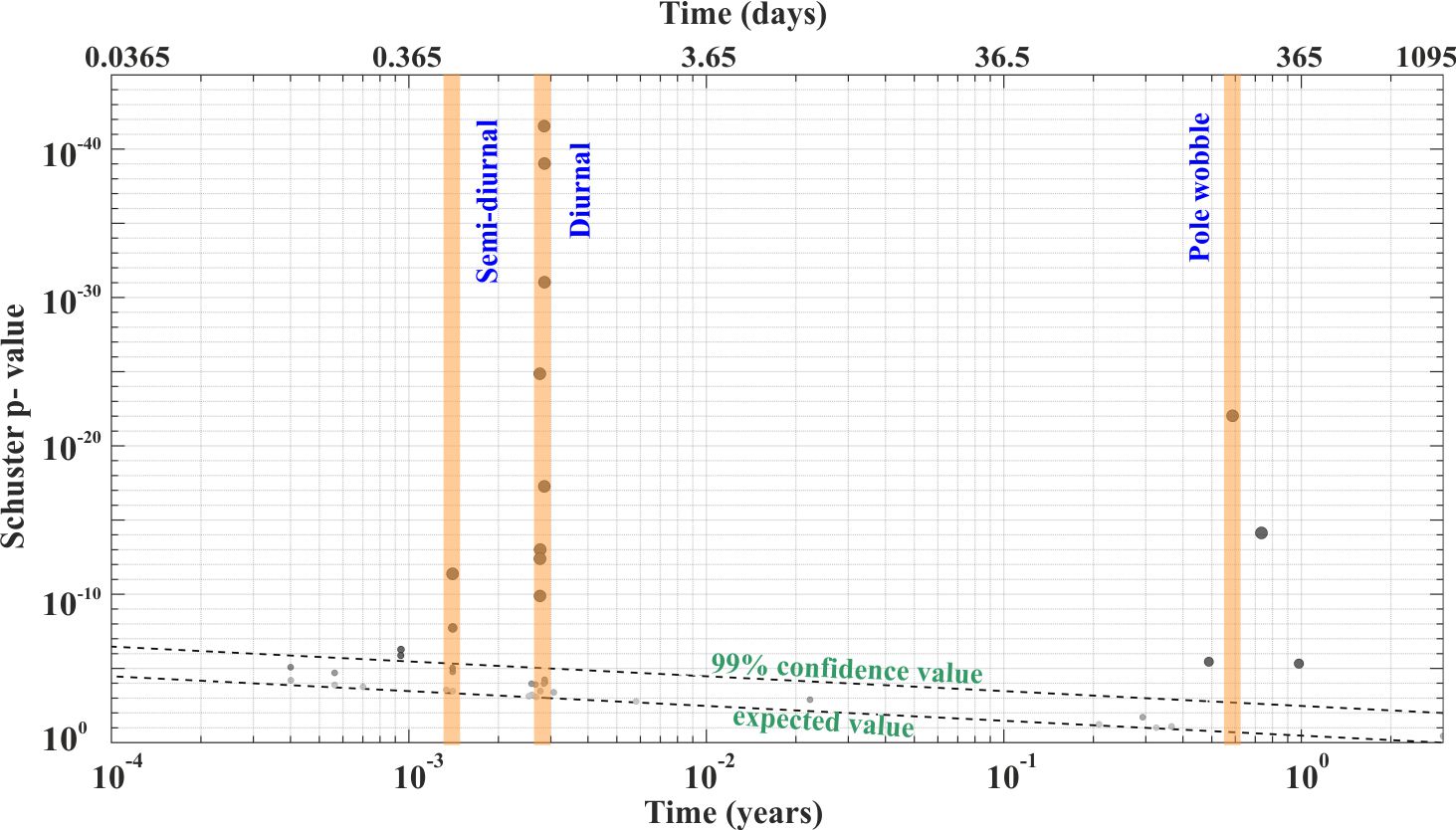


**Figure. 15:** Schuster spectra analysis of Marsquakes considering the full InSight dataset (1200 days). Note that Marsquakes exhibit strong diurnal, Semi-diurnal, and weak pole wobble periodicity.

**Note:** We have also analysed the periodicity of the Marsquakes, considering the full InSight dataset (1200 days) (Fig. 15). The full InSight dataset (1200 days) is taken from the Dahmen et al., 2022 (JGR: Planets, https://doi.org/10.1029/2022JE007503). From this analysis, we have noticed a prominent well-demarcated peak at diurnal and semi-diurnal periods. We have also noticed another prominent periodicity close to ~238 days, which appears to be fairly close enough to the reported Chandler Wobble of Mars (~206.9 days) (Fig. 15).


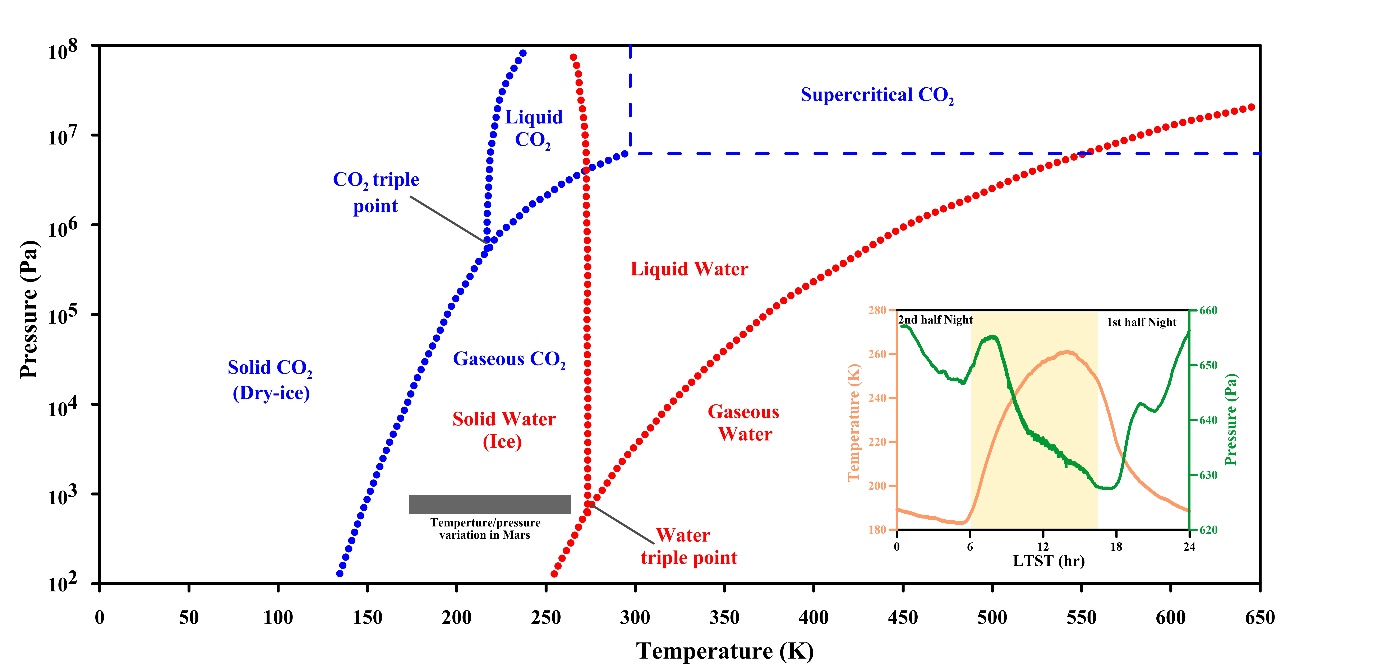


**Figure S16.** Phase diagram for water and carbon dioxide. The grey bars represent the variation of temp and pressure in the mars surface. The inset shows the variation of temperature and pressure in the Mars surface.

**Note:** The pressure and temperature data of Mars are recorded by the Auxiliary Payload Sensor Subsystem (APSS), pressure sensor and the temperature and wind (TWINS) sensor of the Mars Insight, which are available at the PDS Atmospheres node and archived at the <https://atmos.nmsu.edu/data_and_services/atmospheres_data/INSIGHT/insight.html#Selecting_Data>. The daily pressure and temperature variation on Mars is about 40 Pa and 80°C (inset in Fig. S15). This diurnal variation in atmospheric pressure and temperature in Mars is plotted in the P-T stability phases of water (or ice) and carbon dioxide (or dry ice) (grey bar in Fig. S15). From this graph, it is observed that during the day to night time the Carbon dioxide present in Mars is stable in the Gaseous phase, although during night time it approaches the dry-ice phase, it is not cross the stability boundary between the solid and gaseous phase (Fig. S15).

**Table S1.** Gravitational acceleration of the Solar System objects:

| **Planetary body** | **Gravity (g, m/s^2^)** | $\frac{\boldsymbol{g}}{\boldsymbol{g}_{\boldsymbol{Earth}}}$ |
| --- | --- | --- |
| Sun | 274.13 | 27.95 |
| Mercury | 3.59 | 0.37 |
| Venus | 8.87 | 0.90 |
| Earth | 9.81 | 1.00 |
| Moon | 1.62 | 0.17 |
| Mars | 3.77 | 0.38 |
| Jupiter | 25.95 | 2.65 |
| Saturn | 11.08 | 1.13 |
| Uranus | 10.67 | 1.09 |
| Neptune | 14.07 | 1.43 |
| Pluto | 0.42 | 0.04 |

**Table S2.** Seismicity modulation by the external stress perturbation observed in plate boundary and plate interior setting of Earth

| **Plate interior** | **Regions** | **Periodicity** | **Remarks** | **References** |
| --- | --- | --- | --- | --- |
|  | Koyana-Warna, India | Annual | Reservoir induced | Yadav et al.^19^ |
|  | Teheri, India | Annual | Reservoir induced | Chander and Gahalaut^20^ |
|  | Delhi, India | Semi-annual | Groundwater | Tiwari et al.^9^ |
|  | New Madrid Seismic zone, USA | Annual | Hydrological load induced | Craig et al.^10^ |
|  | Aswan, Egypt | Annual | Reservoir induced | Gahalaut et al.^21^ |
| **Plate boundary** | Nankai subduction zone | Semi-diurnal, diurnal | Tidal induced | Rubinstein et al.^22^ |
|  | Cascadia subduction zone | Annual, semi-diurnal, diurnal, pole tide | Hydrological load and Tidal induced | van der Elst et al.^23^ |
|  | Chile subduction zone | semi-diurnal, diurnal | Tidal induced | Gallego et al.^24^ |
|  | Alpine fault | Annual | Hydrological load induced | Oestreicher^25^ |
|  | Nepal Himalaya | Annual | Hydrological load induced | Kundu et al.^26^ |
|  | Juan de Fuca ridge | semi-diurnal | Tidal induced | Sahoo et al.^31^ |
|  | East Pacific rise | semi-diurnal, diurnal | Tidal induced | Tan et al.^27^ |
|  | Guerrero subduction zone | Annual | Hydrological load induced | Lowry^28^ |
|  | East Africa rift | Annual | Hydrological load induced | Xue et al.^29^ |
|  | Mexico & Bungo channel | pole tide | Tidal induced | Shen et al.^30^ |
|  | Taiwan | Fortnight | Tidal induced | Chen et al.^31^ |

**Table S3.** Seismicity modulation by the external stress perturbation on Moon.

| **Shallow**  **Moonquakes** | **Periodicity** | **Remarks** | **References** |
| --- | --- | --- | --- |
|  | 29.5 days | Thermal induced | Dimech et al.^2^; George and Sutton^32^ |
|  | 27 days (?) | Tidal induced | Watters et al.^33^ |
| **Deep**  **Moonquakes** | 27 days, 13.6 days | Tidal induced | Lammlein^34^ |
|  | 206 days and 6 year (?) | Tidal effect from sun | Lammlein^35^ |

**Table S4.** Physical parameters used for thermoelastic stress computation for thermal Moonquakes:

| Thermal diffusivity (κ) | 5 × 10^-7^ m^2^/s | Tanimoto et al.^36^ |
| --- | --- | --- |
| Poisson’s ratio (ν) | 0.25 | Lu et al.^37^ |
| Thermal expansion coefficients (β) | 3 × 10^-7^ °C^-1^ | Horai et al.^38^ Tanimoto et al.^37^ |
| Young’s modulus (E) | 5 × 10^9^ N/m^2^ | Horai et al.^38^; Tanimoto et al.^36^ |
| Spatial wavelength (λ) | 3, 10 km | Lu et al.^37^ |

**Table S5.** Seismicity modulation by the external stress perturbation observed on Mars

| **Marsquakes** | **Periodicity** | **Remarks** | **References** |
| --- | --- | --- | --- |
|  | Annual | Seasonal modulation | Knapmeyer et al.^4^ |
|  | Diurnal and Semi-diurnal | detection probabilities during daytime | --------------- |
|  | Phobus tide | Possible artifacts | --------------- |
|  | Pole wobble | Chandler wobble of mars | --------------- |

**Supporting references:**

1. F. Waldhauser, Wilcock, W.S.D., Tolstoy, M., Baillard, C., Tan, Y.J., Schaff, D.P., Precision seismic monitoring and analysis at axial seamount using a real-time double difference system. *J. Geophys. Res. Solid Earth* **125** (5) (2020). <https://doi.org/10.1029/2019JB018796>
2. J.L. Dimech, B. Knapmeyer-Endrun, D. Phillips, R.C. Weber, Preliminary analysis of newly recovered Apollo 17 seismic data. *Results in physics*, **7**, 4457-4458 (2017). <https://doi.org/10.1016/j.rinp.2017.11.029>
3. J.F. Clinton, S. Ceylan, M. van Driel, D. Giardini, S.C. Stähler, M. Böse, C. Charalambous, N.L. Dahmen, A. Horleston, T. Kawamura, A. Khan, The Marsquake catalogue from InSight, sols 0–478, *Phys. Earth Planet. Inter.,* **310**, 106595 (2021). <https://doi.org/10.1016/j.pepi.2020.106595>
4. M. Knapmeyer, S.C. Stähler, I. Daubar, F. Forget, A. Spiga, A., T. Pierron, B. Banerdt, Seasonal seismic activity on Mars. *Earth Planet. Sci. Lett,* ***576***, 117171 (2021). <https://doi.org/10.1016/j.epsl.2021.117171>
5. K. Aki, Maximum likelihood estimate of b in the formula logN=a−bM and its confidence limits. Bull. Earthq. *Res. Inst., Tokyo Univ.* **43**, 237–239 (1965).
6. P. Reasenberg, Second-order moment of Central California seismicity, 1969-1982. *J. Geophys. Res*. **90**, 5479–5495 (1985). <https://doi.org/10.1029/JB090iB07p05479>
7. T.J. Ader, J.P. Avouac, Detecting periodicities and declustering in earthquake catalogs using the Schuster spectrum, application to Himalayan seismicity. *Earth & planet. Sci. Lett.,* **377**,97-105 (2013). <https://doi.org/10.1016/j.epsl.2013.06.032>
8. P. Stoica, and Moses, R.L., Spectral analysis of signals (2005).
9. D.K. Tiwari, Jha, B., Kundu, B., Gahalaut, V.K., Vissa, N.K., Groundwater extraction-induced seismicity around Delhi region, India. *Sci Rep.* **11**, 10097 (2021). <https://doi.org/10.1038/s41598-021-89527-3>
10. S.T. Bisrat, H.R. DeShon, C.A. Rowe, Swarm activity within the New Madrid seismic zone identified using waveform cross correlation techniques. *Bull. Seismol. Soc. Am.* **102**, 1167– 1178 (2012). <https://doi.org/10.1785/0120100315>
11. J.T. Craig, K. Chanard, E. Calais, Hydrologically-driven crustal stresses and seismicity in the New Madrid Seismic Zone, *Nat. Commun*. **8**, 2143 (2017). <https://doi.org/10.1038/s41467-017-01696-w>
12. F.F. Pollitz, Wech, A., Kao, H., Bürgmann, R., Annual modulation of non-volcanic tremor in northern Cascadia. *J. Geophys. Res: Solid Earth*. **118**(5), 2445-2459 (2013). <https://doi.org/10.1002/jgrb.50181>
13. S. Sahoo, Senapati, B., Panda, D., Tiwari, D.K., Santosh, M., Kundu, B., Tidal triggering of micro-seismicity associated with caldera dynamics in the Juan de Fuca ridge. *J. Volcanol. Geotherm. Res.* **417**, 107319 (2021). <https://doi.org/10.1016/j.jvolgeores.2021.107319>
14. K. Shiomi, Matsubara, M., Ito, Y., & Obara, K., Simple relationship between seismic activity along Philippine Sea slab and geometry of oceanic Moho beneath southwest Japan. *Geophys. J. Int.* **173**, 1018–1029 (2008). <https://doi.org/10.1111/j.1365-246X.2008.03786.x>
15. B. Senapati, Kundu B, Jin S, Seismicity modulation by external stress perturbations in plate boundary vs. stable plate interior. *Geosci. Front.* **13**,101352 (2022). <https://doi.org/10.1016/j.gsf.2022.101352>
16. S. Kodaira, T. Iidaka, A. Kato, Jin-Oh Park., T. Iwasaki, Y. Kaneda, (2004). High Pore Fluid Pressure May Cause Silent Slip in the Nankai Trough. *Science* **304** (5675), 1295-1298(2004). <https://doi.org/10.1126/science.1096535>
17. V.K. Gahalaut, K. Gahalaut, J.K. Catherine, K.M. Sreejith, R. K.Yadav, C. Mohanalakshmi, H.Naidu,. & V. R. Rao, Geodetic Constraints on Tectonic and Anthropogenic Deformation and Seismogenesis of Koyna–Warna Region. India. *Bull. Seismol. Soc. Am.* **108** (5B), 2933-2942 (2018). <https://doi.org/10.1785/0120170373>
18. K. Gahalaut, V.K. Gahalaut, B. Naresh, M. Shekar, T.C. Sunilkumar, D. Srinagesh, Long duration non-volcanic and non-tectonic Palghar earthquake swarm in the stable continental region of India—role of seasonal rainfall and earthquake cascading. *J. Seismol.,* **26**, 545–554 (2022). <https://doi.org/10.1007/s10950-022-10087-8>
19. A. Yadav, Gahalaut, K., Mallika, K., Rao, N.P., Annual Periodicity in the Seismicity and Water Levels of the Koyna and Warna Reservoirs, Western India: A Singular Spectrum Analysis. *Bull. Seismol. Soc. Am.* **105**(1) 464–472 (2015). <https://doi.org/10.1785/0120140234>
20. A. Chander, K. Gahalaut, Probable influence of Tehri reservoir on earthquakes of Garhwal Himalaya. *Current science*. **70**, 4 (1996). <https://www.jstor.org/stable/24097316>
21. K. Gahalaut, A. Hassoup, H. Hamed, B. Kundu, V.K. Gahalaut, Long-Term and Annual Influence of Aswan Reservoir (Egypt) on the Local Seismicity: A Spatio-Temporal Statistical Analysis. *Pure Appl. Geophys****.* 174**, 133–150 (2017) <https://doi.org/10.1007/s00024-016-1397-6> .
22. J.L. Rubinstein, Mario La Rocca, M.L., John E. Vidale, J.E., Creager, K.C., Wech, A.G., et al., Tidal Modulation of Nonvolcanic Tremor. *Science* **319**, 186 (2008). <https://doi.org/10.1126/science.1150558>
23. N.J. van der Elst, Delorey, A.A., Shelly, D.R., Johnson, P.A., Fortnightly modulation of San Andreas tremor and low-frequency earthquakes. *Proc. Natl. Acad. Sci. U.S.A*. **113** (31), 8601–8605 (2016). <https://doi.org/10.1073/pnas.1524316113>
24. A. Gallego, R.M. Russo, D. Comte, V. Mocanu, R.E. Murdie, J.C. VanDecar, Tidal modulation of continuous nonvolcanic seismic tremor in the Chile triple junction region. *Geochem. Geophys. Geosyst.* **14**, 851–863 (2013). <https://doi.org/10.1002/ggge.20091>
25. N.K. Oestreicher,. Geodetic, hydrologic and seismological signals associated with precipitation and in ltration in the central Southern Alps, New Zealand. *M.S thesis Victoria University of Wellington* (2018).
26. B. Kundu, N.K. Vissa, N. K., Panda, D., Jha, B., Asaithambi, R., Tyagi, B., S. Mukherjee, Influence of a meteorological cycle in mid-crustal seismicity of the Nepal Himalaya. *J. Asian Earth Sci.* **146**, 317-325 (2017). <https://doi.org/10.1016/j.jseaes.2017.06.003>
27. Y.J. Tan, Tolstoy, M., Waldhauser, F., Bohnenstiehl, D. R., Tidal triggering of microearthquakes over an eruption cycle at 9∘50’N East Pacific Rise. *Geophys. Res. Lett.* **45**, 1825–1831 (2018). <https://doi.org/10.1002/2017GL076497>
28. A R Lowry, Resonant slow fault slip in subduction zones forced by climatic load stress. *Nature* **442**, 802–805 (2006). <https://doi.org/10.1038/nature05055>
29. L. Xue, Johnson, Christopher W., Fu,Y., Bürgmann, R., Seasonal seismicity in the Western Branch of the East African Rift System. *Geophys. Res. Lett.* **47**(6), e2019GL085882 (2020). <https://doi.org/10.1029/2019GL085882>
30. Z-K. Shen, Wang, Q., Bu¨rgmann, R., Wan, Y., Ning, J., Pole-Tide Modulation of Slow Slip Events at Circum-Pacific Subduction Zones. *Bull. Seismol. Soc. Am*. **95**, 5, 2009–2015 (2005). <https://doi.org/10.1785/0120050020>
31. K.H. Chen, H.J. Tai, S. Ide, T.B. Byrne, C.W. Johnson, Tidal modulation and tectonic implications of tremors in Taiwan. *J. Geophys. Res.: Solid Earth*, 1**23**(7), 5945-5964 (2018). <https://doi.org/10.1029/2018JB015663>
32. F. Duennebier, G.H. Sutton, Thermal moonquakes. *Geophys. Res. Lett.,* **79**(29), 4351-4363 (1974). <https://doi.org/10.1029/JB079i029p04351>
33. T.R. Watters, Weber, R.C., Collins, G.C. *et al.* Shallow seismic activity and young thrust faults on the Moon. *Nat. Geosci.* **12**, 411–417 (2019). <https://doi.org/10.1038/s41561-019-0362-2>
34. D. R. Lammlein, Lunar seismicity and tectonics, *Phys. Earth Planet. Inter.,* **14**, 224–273 (1977). <https://doi.org/10.1016/0031-9201(77)90175-3>
35. Lammlein, D. R., G. V. Latham, J. Dorman, Y. Nakamura, and M. Ewing, Lunar seismicity, structure and tectonics*, Rev. Geophys.,* **12**, 1–21(1974). <https://doi.org/10.1029/RG012i001p00001>
36. T. Tanimoto, Eitzel, M., and Yano, T., The noise cross‐correlation approach for Apollo 17 LSPE data: Diurnal change in seismic parameters in shallow lunar crust. *J Geophys Res : Planets*, **113**(E8) (2008). <https://doi.org/10.1029/2007JE003016>
37. Z. Lu, L. Wen, Abnormally strong daily‐cycle S1 strain tide: Observation and physical mechanism. *J Geophys Res : Solid Earth,* **122**(10), 8525-8537 (2017). <https://doi.org/10.1002/2017JB014383>
38. K.I. Horai, G. Simmons, H. Kanamori, D. Wones, Thermal diffusivity and conductivity of lunar material. *Science*, **167**(3918), 730-731 (1970). <https://doi.org/10.1126/science.167.3918.730>
